# Heterogeneity and Convergence of Olfactory First-Order Neurons Account for the High Speed and Sensitivity of Second-Order Neurons

**DOI:** 10.1371/journal.pcbi.1003975

**Published:** 2014-12-04

**Authors:** Jean-Pierre Rospars, Alexandre Grémiaux, David Jarriault, Antoine Chaffiol, Christelle Monsempes, Nina Deisig, Sylvia Anton, Philippe Lucas, Dominique Martinez

**Affiliations:** 1 Institut National de la Recherche Agronomique (INRA), Unité Mixte de Recherche 1392 Institut d'Ecologie et des Sciences de l'Environnement de Paris, Versailles, France; 2 Laboratoire Lorrain de Recherche en Informatique et ses Applications (LORIA), Unité Mixte de Recherche 7503, Centre National de la Recherche Scientifique (CNRS), Vandœuvre-lès-Nancy, France; Duke University, United States of America

## Abstract

In the olfactory system of male moths, a specialized subset of neurons detects and processes the main component of the sex pheromone emitted by females. It is composed of several thousand first-order olfactory receptor neurons (ORNs), all expressing the same pheromone receptor, that contact synaptically a few tens of second-order projection neurons (PNs) within a single restricted brain area. The functional simplicity of this system makes it a favorable model for studying the factors that contribute to its exquisite sensitivity and speed. Sensory information—primarily the identity and intensity of the stimulus—is encoded as the firing rate of the action potentials, and possibly as the latency of the neuron response. We found that over all their dynamic range, PNs respond with a shorter latency and a higher firing rate than most ORNs. Modelling showed that the increased sensitivity of PNs can be explained by the ORN-to-PN convergent architecture alone, whereas their faster response also requires cell-to-cell heterogeneity of the ORN population. So, far from being detrimental to signal detection, the ORN heterogeneity is exploited by PNs, and results in two different schemes of population coding based either on the response of a few extreme neurons (latency) or on the average response of many (firing rate). Moreover, ORN-to-PN transformations are linear for latency and nonlinear for firing rate, suggesting that latency could be involved in concentration-invariant coding of the pheromone blend and that sensitivity at low concentrations is achieved at the expense of precise encoding at high concentrations.

## Author Summary

Understanding how sensory signals are optimally encoded by nervous systems is of strong interest to neuroscientists, and also to engineers as it may lead to more efficient artificial detection systems. This is particularly relevant to olfaction, because the current electronic noses are far outperformed by their biological counterparts in terms of speed and sensitivity. We here use the moth sex pheromone processing system as a relatively simple model to understand early olfactory coding. We found that performance increases when olfactory information passes from first- to second-order neurons. Second-order neurons respond on average with shorter latency and higher sensitivity than first-order neurons. We show that two critical factors, convergent architecture and neuronal heterogeneity, are needed to account for increased performance.

## Introduction

In insects and vertebrates the first two neuronal layers of the olfactory system present the same organization where many ORNs in the first layer converge to a small number of output neurons in the second layer – PNs in insects and mitral cells in vertebrates [1], [2]. The ORNs that project onto a single glomerulus express a single type of olfactory receptors. Yet, they present heterogeneous dose–response properties [3].

The functional consequence of this convergence has been the subject of much interest. Theory [4] predicts and experiments [5] confirm that pooling *N* ORN inputs should increase the firing rate of output neurons by *N* and improve the signal-to-noise ratio by *√N*. Experiments in the fruit fly reveal that firing rates rise more rapidly in PNs than in ORNs and that weak odor inputs are more amplified than strong inputs [6]-[8]. Such a non-linear transformation leads to an efficient use of coding capacity [6] and a maximum preservation of information on odor quality [9].

Although previous studies investigated the change in firing rate when sensory information passes from first- to second-order neurons, they did not consider the latency of the response. This is restrictive given that environmental conditions put strong constraints on the behavioral response time. In natural odor plumes, encounters with the stimulus are brief and intermittent, with up to five contacts per 1 s and each contact lasting down to under 20 ms [10]–[12]. Consequently, behavioral responses to odors are fast (<100 ms in rodents [13]–[15] and insects [16], [17]) and the olfactory system, like other sensory systems [18], [19], may rely on response latencies for fast odor discrimination [10], [12], [20].

In this work, we compared the transformations in latency and firing rate from first- to second-order neurons and assessed whether the cell-to-cell heterogeneity contributes to this transformation. We addressed these issues in a favorable model, the male moth subsystem that processes the sex pheromone. Each pheromone component activates with high specificity a single ORN type [5] whose axons project to one of a few glomeruli – the macroglomerular complex (MGC). The second-order neurons (PNs and local neurons LNs) in the largest MGC glomerulus – the cumulus – receive their sole olfactory input (homotypic) from the most abundant ORN type sensitive to the major component, with no lateral olfactory input from other glomeruli (heterotypic) (e.g. [21], [22]). This is a significant advantage for experimental analysis with respect to glomeruli sensitive to general odors that receive both homotypic and heterotypic inputs because of the lack of specificity of generalist (non-pheromonal) ORNs [7]. Apart from this difference, which allowed us to record responses with well-defined input, the isomorphic glomeruli involved in general odor processing and the cumulus share the same functional organization, so that the main properties found here for the pheromonal system should apply also to the general odorant system.

We found that both firing rates and latencies of ORNs and PNs are strongly dose-dependent, that PNs respond with a higher firing rate and a shorter latency than most ORNs, and that the sensitivity and speed of a given neuron are not correlated. We found also that dose-response curves are variable among ORNs (and PNs) and that this variability of essentially biological origin arises more from the variability across neurons (heterogeneity) than within single neurons (irregularity). For firing rate, the ORN-to-PN amplification mechanism is non-linear, which augments sensitivity to weak odor signals in intensity coding and enhances distinction between different general odors in quality coding [6]; it can be explained by the convergence of many ORNs on single PNs. For latencies, on the contrary, the ORN-to-PN transformation is linear, which might favor concentration-invariant coding of odor blends, and requires ORN heterogeneity.

## Results

### Spontaneous and evoked activities

**Figure 1:**
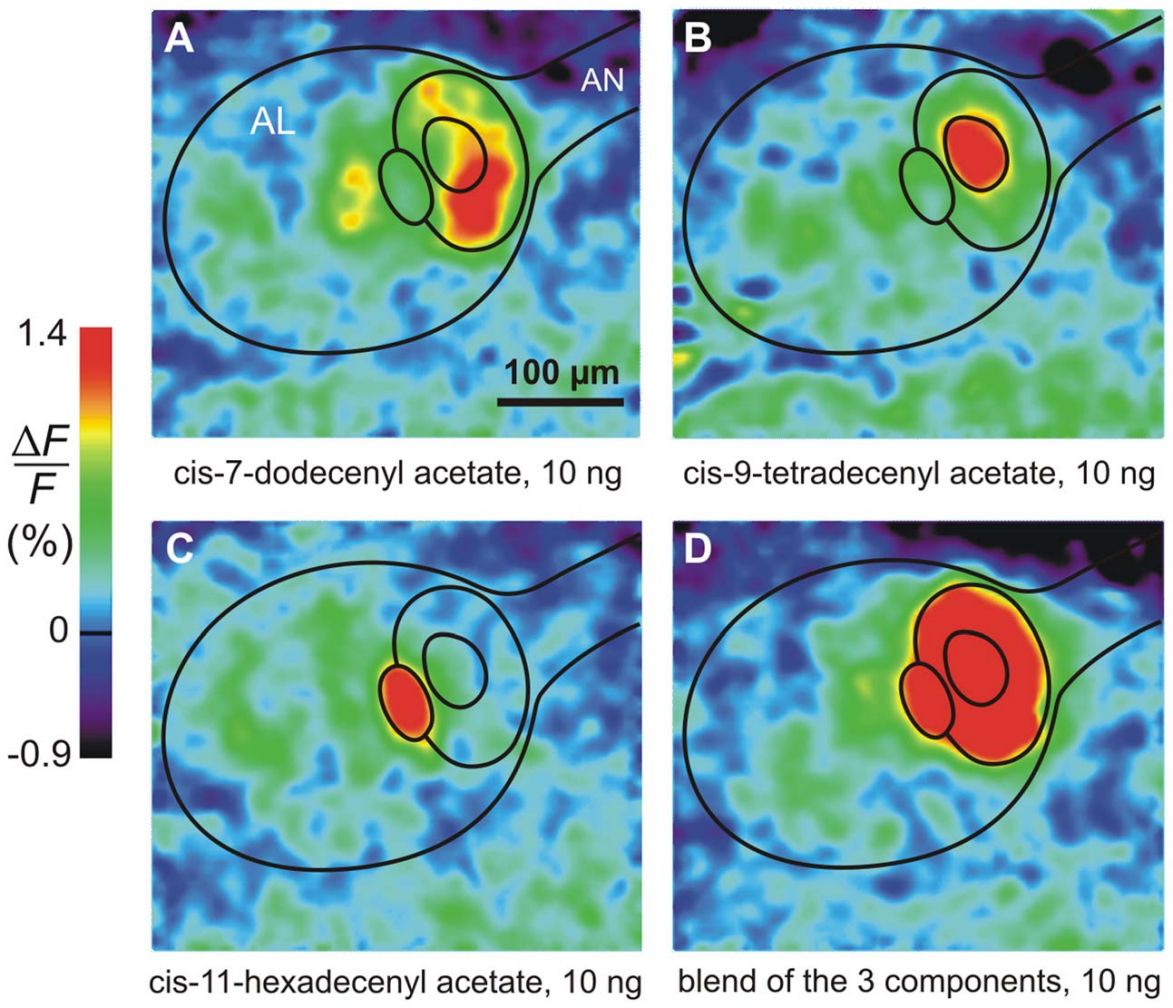
Ca^2+^ imaging shows that each pheromone component activates a single glomerulus in the MGC. (**A**) The main pheromone component activates the cumulus only. (**B, C**) The two secondary components activate two neighboring glomeruli. (**D**) The blend of the 3 components in the behaviorally most efficient ratio 4∶1∶4 activates the whole MGC. Outlines of antennal lobe (AL), antennal nerve (AN) and the 3 main subdivisions of MGC are shown.

**Figure 2:**
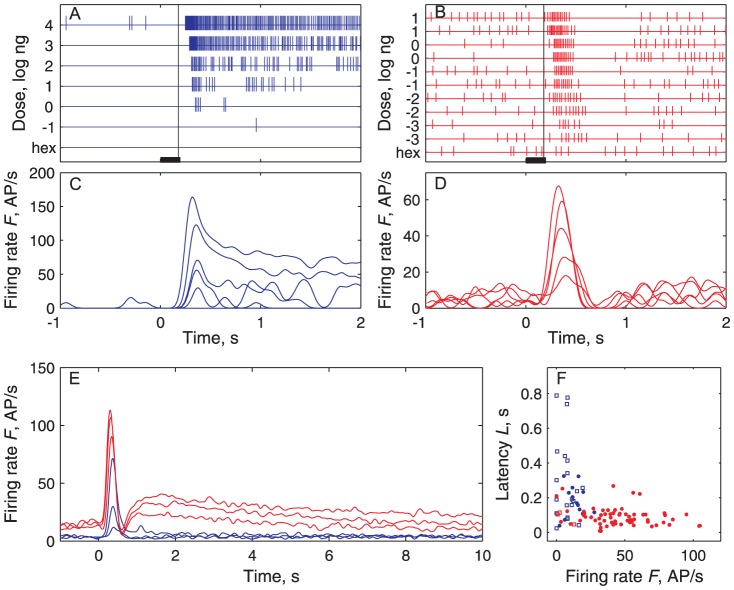
Pheromone-evoked spiking activities are qualitatively and quantitatively different in ORNs and PNs. In this and all following figures ORNs are shown in blue and PNs in red. (**A**) Phasic-tonic activity in a single ORN at various doses *C* of Z7-12∶Ac from -1 to 4 log ng (bar: stimulus duration, 200 ms). Schematic representation based on spike sorting. Hexane (hex) used as control. Vertical line at *T*_t_ = 180±13 ms (mean ± SD) indicates mean time of arrival of stimulus on antenna. (**B**) Multiphasic activity in a PN at doses from -3 to 1 with repetitions. Same representation as in (A). (**C**) Instantaneous firing rates estimated with a 50 ms Gaussian kernel (see Methods) of spike trains shown in (A). (**D**) Instantaneous firing rates of the trains shown in (B). (**E**) Comparison of average instantaneous firing rates of ORNs and PNs recorded at doses -1, 0 and 1 log ng. (**F**) Firing rate *F* versus latency *L* pairs from the same pheromone-evoked response for all ORNs (blue) and PNs (red) recorded at dose *C* = −1 log ng (responses significantly different shown as filled circles; all other figures show only responses significantly different from spontaneous activity).

When stimulated with the components of the pheromone, the cumulus of the male moth *Agrotis ipsilon* was activated only by the main pheromone component, cis-7-dodecenyl acetate (Z7-12:Ac) (Fig. 1A). Conversely, the other glomeruli in the MGC were activated only by the other pheromone components (Fig. 1B–C). In electrophysiological recordings, the Z7-12:Ac-responsive ORNs displayed phasic-tonic responses (Fig. 2A, C) whereas the second-order neurons we studied shared a common multiphasic response pattern with an initial excitation followed by an inhibition (Fig. 2B, D) and frequently a final rebound (Fig. 2E). All stained multiphasic neurons were found to be PNs with dendritic trees in the cumulus and axons in the inner antenno-cerebral tract. The rare stained LNs we found (3 among 67 stained cells) were monophasic. Although these observations do not rule out the existence of LNs with a multiphasic response pattern, they support the contention that multiphasic LNs (if they exist) are rare in our recording conditions, which means that most if not all recorded neurons were PNs. For this reason, in the following, we used the more common term PN.

**Figure 3:**
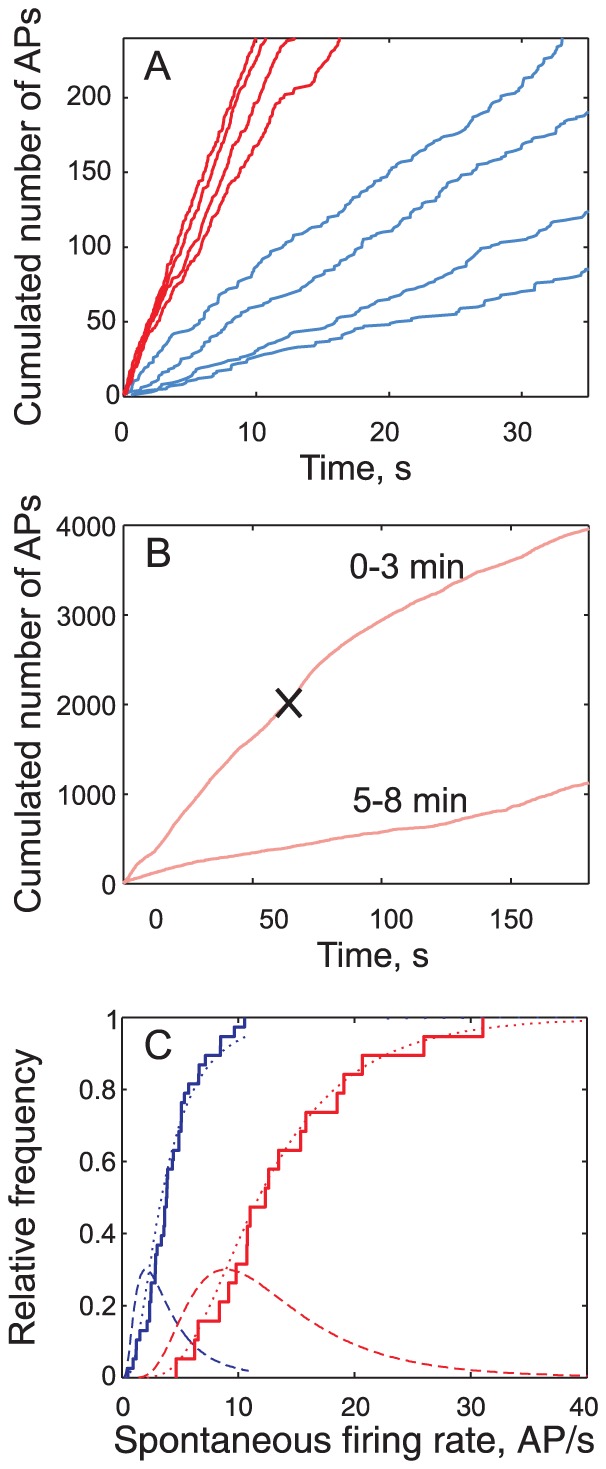
Spontaneous activity in PNs is higher than in ORNs and depends on ORN spontaneous activity. (**A**) Total number of spontaneous spikes *N*_sp_ fired from time 0 to *t_i_* (firing time of *i*th spike) plotted as a function of *t_i_* in 4 ORNs (blue) and 4 PNs (red). The mean spontaneous firing rate is the slope of the regression line of *N*_sp_ vs. *t*. (**B**) Spontaneous activity of a PN before and after sectioning the antennal nerve (black cross); same representation as in (A). Top curve: first 3 min with sectioning marked with cross; slope of regression line before sectioning  = 32 AP/s. Bottom curve: same neuron from 5 to 8 min after sectioning (slope  = 5.6 AP/s). (**C**) Distribution of spontaneous firing rates in ORNs (blue) and PNs (red), with empirical cumulative distribution functions (CDFs, staircase), fitted lognormal CDFs (dashed curve) and corresponding probability distribution functions (PDFs, dotted curve). Parameters of these distributions are given in S2 Table.

Even in the absence of pheromone delivery, the Z7-12:Ac-responsive ORNs and PNs spiked tonically. This spontaneous activity is stationary (Fig. 3A) with a median firing rate lower in ORNs than in PNs (Fig. 3A, C). The distributions of spontaneous firing rates *F*_sp_ (all symbols are defined in S1 Table) are well fitted to lognormal distributions with a longer tail in PNs than in ORNs (Fig. 3C; S2 Table). To determine whether the PN activity is influenced by ORNs at rest, the antenna was sectioned. The PN firing rate began to decrease ∼10 s after the section and reached a stable regime (∼70% lower, range 58-85%) after less than 5 min (Fig. 3B).

The present study is restricted to two aspects of the olfactory code – the initial firing rate *F* (as defined in S1B, D Figure) and response latency *L* (S1A, B Figure) and their dose-dependent transformations – without ignoring that other aspects of the responses, e.g. action potentials after stimulus offset [23] or correlated activity in different neurons [24], may also provide useful information.

### A paradox in response latencies

A feature of the studied response variables is immediately noticeable. The pairs (*F*, *L*) of a given response recorded from ORNs and PNs are quite distinct, especially at low doses. For example at dose *C* = −1 log ng, PNs fire with higher rates and shorter latencies than ORNs so that most pairs (*F*, *L*) from ORNs and PNs do not overlap (Fig. 2F). This may seem paradoxical because one would expect that the shortest latencies be a little longer in PNs than in ORNs on account of axonal conduction and synaptic transmission. This apparent paradox is the main theme of this paper and its resolution required to analyze how the neuron responses depend (or not) on the dose, the ORN and PN variability and the ORN-to-PN convergence ratio.

**Figure 4:**
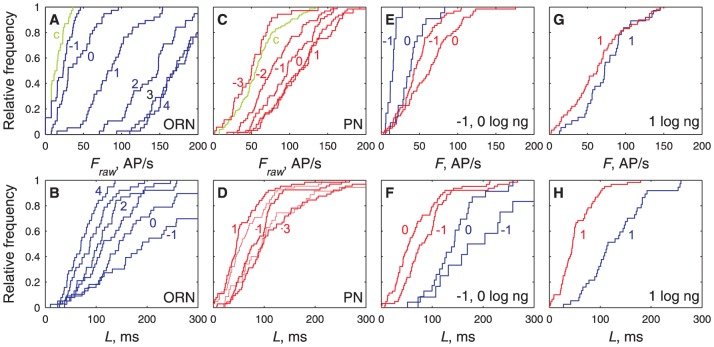
Distributions of firing rates (top row) and latencies (bottom row) at single pheromone doses are dose-dependent. (**A**) Comparison in ORNs of raw firing rates *F*_raw_ (not corrected from control stimulations) for control stimulations (green) and for pheromone doses −1, 0, 1, 2, 3, 4 log ng (blue, from left to right). *F*_raw_ at *C* = −1 log ng not significantly different from control (Kolmogorov-Smirnov test, *p* = 0.43). (**B**) Comparison in ORNs of latencies *L* for same stimuli and doses (from right to left) as in (A). (**C**) Comparison in PNs of firing rates *F*_raw_ for control stimulations (green) and for pheromone doses −3, −2, −1, 0, 1 log ng (red), same representation as in (A). *F*_raw_ at *C* = −3 log ng not significantly different from control (Kolmogorov-Smirnov test, *p* = 0.43) but significantly different from *F*_raw_ at *C* = −2 (*p*<10^−4^). (**D**) Comparison in PNs of latencies *L* for same stimuli and doses as in (C). (**E, G**) Comparison of firing rates *F* (corrected from control stimulation) in ORNs (blue) and PNs (red) at the same doses −1, 0 (in E) and 1 log ng (in G). For *C*≤1, the mean firing firing rates of ORNs is smaller than that of PNs. (**F–H**) Comparison of latencies, same representation as in (E, G). At all doses, the mean firing latency of ORNs is larger than that of PNs. At *C*≥1, the shortest ORN latencies become almost as short as the shortest PN latencies.

**Figure 5:**
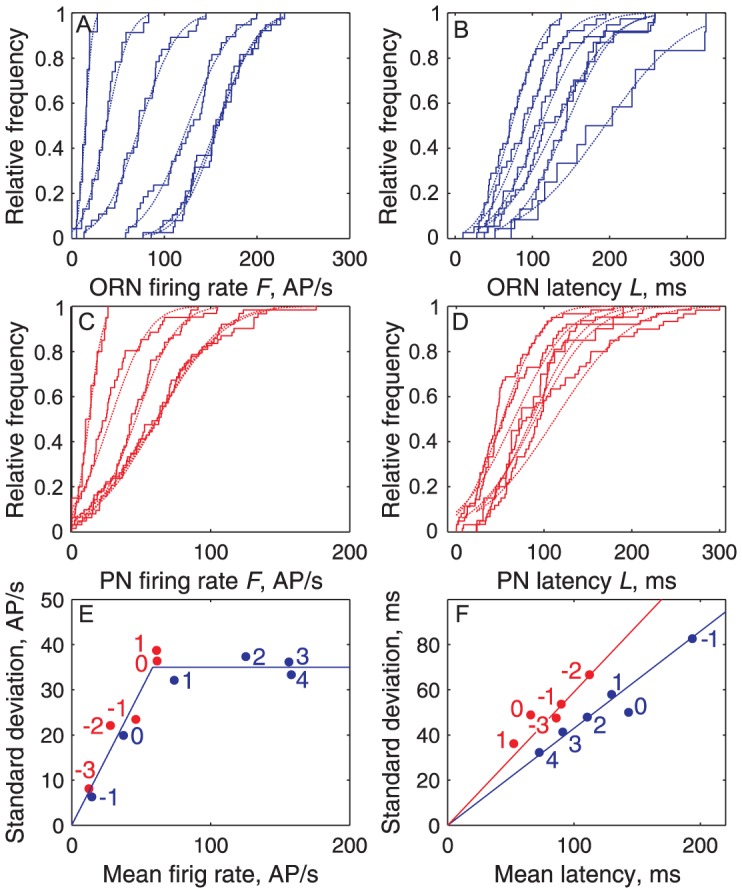
Firing rates and latencies at single pheromone doses are normally distributed with standard deviations related to means. (**A**) Empirical CDFs of firing rates *F* (corrected from control stimulations) for ORN significant responses (staircases) from *C* = −1 (left) to 4 log ng (right) and fitted Gaussian distributions (dashed lines) of means *F*_μ_ and standard deviations *σ*_Tr_. (**B**) Same as (A) for ORN latencies. (**C**) Same as (A) for PNfiring rates. (**D**) Same as (A) for PN latencies. (**E**) Response heterogeneity plot of *σ*_Tr_ versus *F*_μ_ as determined in (A) for ORNs (blue dots) and in (C) for PNs (red dots), with regression lines *σ*_Tr_ ≈0.331 *F*_μ_ AP/s for *C* = −1 to 1 log ng (ORNs, dose indicated in blue) and −3 to −1 (PNs, in red) and *σ*_Tr_ ≈35 AP/s for *C*>1 (ORNs) and> −1 (PNs). (**F**) Response heterogeneity plot of σ_Tr_ versus *L*_μ_ for ORNs (blue) and PNs (red) based on (B, D).

In order to document this feature and to provide an overview of how the two neuron populations studied respond to a given dose of Z7-12:Ac the firing rates and latencies of all recorded neurons at each applied dose were pooled. It was found in this way that the firing rate *F* presents four distinct properties (Fig. 4, top row; Fig. 5, left column): (i) The firing rates across neurons stimulated at the same dose follow Gaussian distributions in ORNs (Figs. 4A, 5A) and PNs (Figs. 4C, 5C). (ii) The mean of the distributions increases with the dose (Fig. 5A, C). At the lowest dose applied (−1 log ng for ORNs, −3 for PNs) the distributions are not significantly different from the control stimulations with pure air or hexane (Fig. 4A, C). The frequencies at the two highest doses tested (3 and 4 log ng for ORNs, 0 and 1 log ng for PNs) are also not significantly different (Fig. 4A, C). Therefore, when measured at the population level, the dynamic ranges of ORNs extend from −1 to 3 log ng and for PNs from −3 to 0 log ng. (iii) For doses *C*≤1, PNs respond more strongly than ORNs (Fig. 3E, G). (iv) The standard deviation of the distributions increases linearly with their mean for *F*<∼100 AP/s, with the same slope in ORNs and PNs (i.e. same coefficient of variation CV≈0.33), notwithstanding the very different doses evoking the same variability in the two populations (Fig. 5E). Above ∼100 AP/s, before saturation of the mean ORN firing rate, the variability of ORNs and PNs becomes constant (Fig. 5E).

Similarly, the latencies at each dose follow Gaussian distributions (Fig. 5B, D) with standard deviations proportional to their means (Fig. 5F). However, contrary to frequencies, (i) the variability decreases when the dose increases; (ii) the slopes (CVs) for ORNs and PNs are different, indicating a higher variability in PNs (Fig. 5F); (iii) no discontinuity in slope is seen at high doses; and (iv), at the same dose, the PN distribution is always shifted to the left of the ORN distribution (Fig. 4F, H) which shows that, at all doses, the PN latencies are shorter than the ORN latencies, thus confirming that the paradox noted above holds at all doses.

### The range of dose-independent properties is narrower in PNs than in ORNs

However, the mere statistical pooling of all available responses at a given pheromone dose, as done in the previous section, is not sufficient to describe adequately the neuron properties. A more detailed view of the system requires that responses to stimulations at increasing doses be analyzed on individual cells, not only cell populations. The main features of dose-response curves in single neurons are examined: first their overall “shape” in the present subsection, then their location along the dose axis and their correlations in the next subsections.

**Figure 6:**
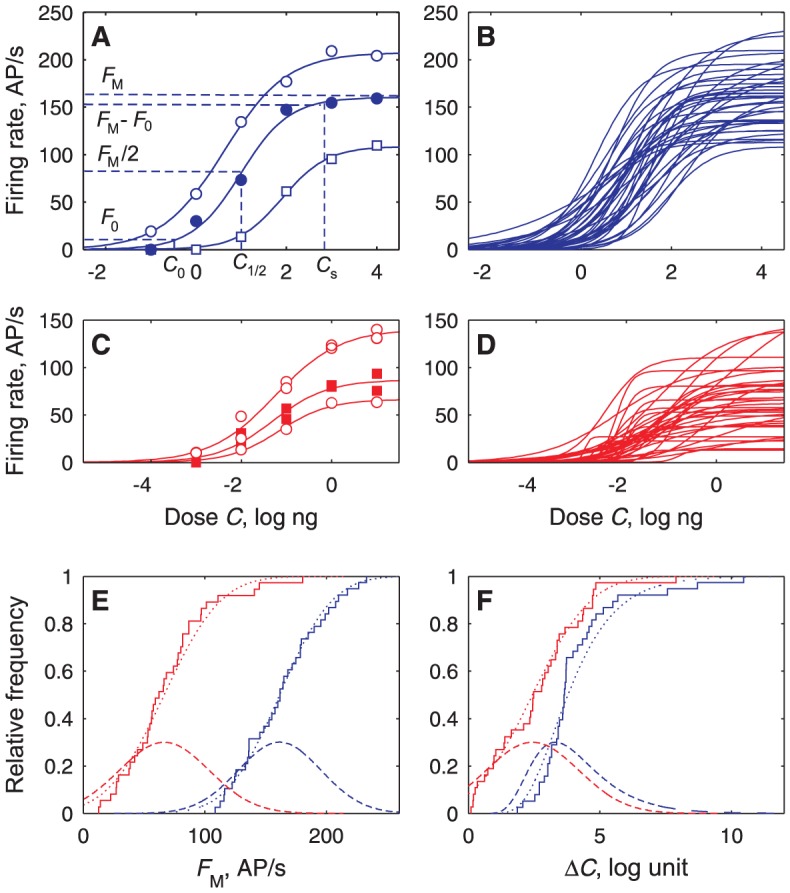
Firing rates are Hill functions of dose with different parameter values in each neuron. (**A**) Measured firing rate *F* (dots) of 3 ORNs fitted to Hill functions (eq. 4; solid curves) showing parameters *F*_M_ and *C*_1/2_ and characteristic *C*_0_ and *C*_s_ for *F*_0_  =  5 AP/s. (**B**) All (*N*  =  38) Hill curves fitted to ORNs. (**C**) Hill curves of 3 PNs. (**D**) All (*N*  =  37) PN curves successfully fitted to Hill functions. (**E**) Distribution of maximum firing rates *F*_M_ in the ORN (blue, *N* = 38) and PN (red, *N* = 37) populations. Each empirical CDF (staircase) with its fitted normal CDF (dotted curve) and corresponding PDF (dashed curve). (**F**) Distributions of dynamic ranges *ΔC* (related to *n*), same *N* and representation as in (E) except fitted distribution is lognormal for ORNs.

**Figure 7:**
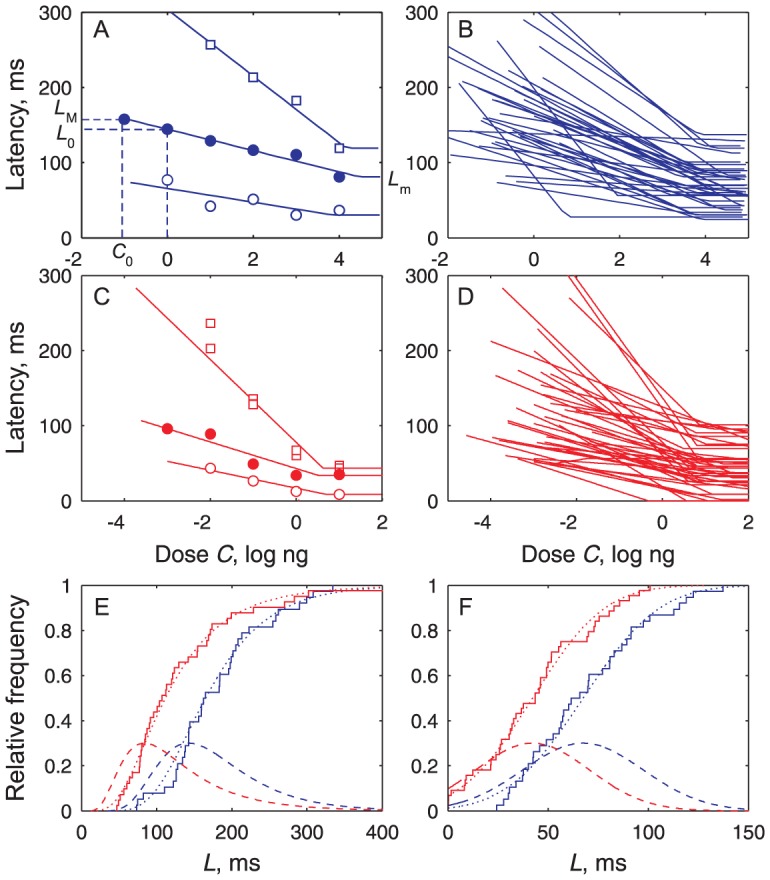
Latencies are linear functions of pheromone dose with different parameter values in each neuron. (**A**) Measured latency *L* (dots) of 3 ORNs fitted to decreasing lines (eq. 8; solid curve) showing minimum latency *L*_m_ and maximum latency *L*_M_ at threshold *C*_0_ given from Fig. 6A. (**B**) All (*N* = 38) fitted ORN dose-latency curves. (**C**) Three examples of PN latency curves. (**D**) All (*N* = 44) fitted PN dose-latency curves. (**E**) Maximum latencies *L*_M_ at threshold dose *C*_0_ fitted to lognormal CDFs; same *N*'s as in (B, D) and representation as in Fig. 6E. (**F**) Minimum latencies *L*_m_ fitted to normal CDFs; same *N* and representation as in (E). A few zero latencies arise in PNs from variability on pheromone transport time *T*_t_.

Dose-response plots were established for 38 ORNs and 47 PNs. Sigmoid Hill functions (see eqs. 3 and 4 in Methods) were fitted to the dose-firing rate *C*-*F* plots (Fig. 6). From the fitted parameters – maximum firing rate *F*_M_, efficient dose 50% (ED50) *C*_1/2_, and Hill coefficient *n* – we also derived three characteristics: the doses at threshold *C*_0_ and at saturation *C*_S_ and their difference, the dynamic range Δ*C* (eqs. 5–7;). The firing rate responses to the lowest doses are not significantly different from controls (Fig. 4A, C) and those to the two highest doses are nearly equal (within 15% in 87% of ORNs and 80% of PNs, showing that the observed maximum firing rates were close to the asymptotic *F*_M_) which guarantees that the parameters were correctly estimated. Latencies were analyzed the same way. Decreasing linear functions with a lower bound were fitted to the dose-latency *C-L* plots (Fig. 7). Each neuron was characterized by its maximum latency *L*_M_ at threshold *C*_0_, its minimum latency *L*_m_ and their difference Δ*L*  =  *L*_M_ − *L*_m_.

Complementary aspects of the distributions of these coding properties such as averages, variability and correlations were analyzed. As far as the “shape” parameters are concerned, we found that the typical ORN, reconstructed from the median values of the fitted parameters (S3 Table), has a high maximum firing rate *F*_M_ (163 AP/s) and a wide dynamic range Δ*C* (3.6 log units), whereas the typical PN, reconstructed in the same way, has a lower *F*_M_ (62 AP/s) and a smaller Δ*C* (2.5 log units). For latencies (S4 Table), the maximum *L*_M_ (164 vs. 107 ms) and the range Δ*L* (104 vs 64 ms) are greater for the typical ORN than the typical PN. Except for *F*_M_, the variability (SD or interquartile range) of all these properties is slightly higher in PNs than in ORNs (Figs. 6E, F and 7E, F).

### Despite narrower properties, PNs are more sensitive and faster than ORNs at the same dose

Although the dose-response curves of ORNs and PNs are basically similar in shape, the essential difference between them is that they are not located identically on the dose axis, which means that ORNs and PNs respond with similar firing rates and latencies but at very different doses. This calls for a clear distinction of the dose-independent properties (like *F*_M_, Δ*C, L*_M_, Δ*L*), analyzed above and the dose-dependent properties (*C*_0_, *C*_1/2_, *C*_s_) which are examined now.

**Figure 8:**
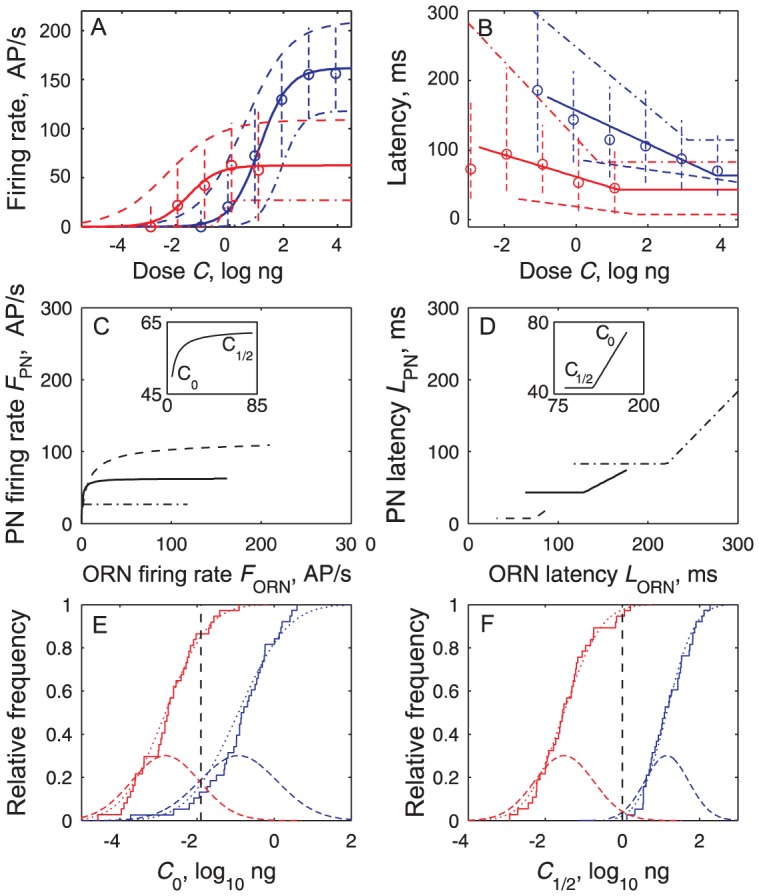
Dose-response curves of PNs are shifted to left of ORN curves and explain ORN-to-PN transfer functions. (**A**) Medians (circles) and quantiles 10% and 90% (vertical dashed lines) of all *F* measured at a given dose, as shown in Fig. 5. Dose-firing rate curves of ORN (blue) and PN (red) populations reconstructed from parameters of individual *C*–*F* curves shown in Fig. 6, based on median (solid), 10% most responsive neurons (dashed, based on quantiles 90% for *F*_M_ and 10% for *C*_1/2_, *n*) and 90% less responsive neurons (dash-dotted). (**B**) Dose-latency curves of ORNs (blue) and PNs (red) based on median, 90% and 10% quantiles. Same representations as in (A) based either on pooled *L* (Fig. 5) or on parameters of *C-L* curves (Fig. 7). (**C**) Median transfer function for firing rates (solid, eq. 9); it can be approximated by *F*_PN_  = 62.5/(1+ (1.5/*F*_ORN_)^1.15^); inset: detail of most nonlinear part from threshold to ED50 of ORNs. Transfer function for the 10% most responsive neurons (dashed, derived from (A) by coupling most responsive ORNs and PNs) and for the 10% least responsive ones (dash-dotted). (**D**) Median transfer function for latencies running from right (low doses) to left (high doses) (solid, eq. 12); inset: linear part from threshold to ED50 of ORNs. Transfer functions for the 10% fastest neurons (dashed) and for the 10% slowest neurons (dash-dot). (**E**) Distributions of thresholds *C*_0_ in ORNs (blue, *N* = 38) and PNs (red, *N* = 37); empirical CDFs (staircases) with fitted normal CDF (solid curve) and corresponding PDF (dashed curve); maximum contrast at *C*_0Δ_  = −1.9 log ng (dashed vertical line) with 17% ORNs and 85% PNs activated. (**F**) Distributions of ED50 *C*_1/2_, same *N*'s and representation as in (E); maximum contrast at *C*_1/2Δ_  = 0 log ng (dashed vertical line) with 2% of ORNs and 98% of PNs above their *C*_1/2_.

PNs are clearly more sensitive than ORNs. For example, the recruitment of PNs starts at lower doses as half of the PNs were activated at −3 log ng and half of the ORNs only at −0.5 log ng (not shown). PNs approach saturation at doses 3 orders of magnitude lower than ORNs. These changes testify that major transformations take place in the neural network of the cumulus when the sensory signal passes from ORN to PN. The ORN-to-PN transformations can be represented in two complementary ways: either by pairs of dose-response curves (Fig. 7A, B), or by transfer functions linking the latencies (or frequencies) of the ORNs and PNs at the same doses (Fig. 8C, D). Interestingly these curves (and the transfer functions derived from them) can be determined in two different ways, either directly from the pooled distributions of *F* and *L* at each dose (Figs. 4, 5), or from the distributions of the parameters determined on single dose-response plots (Figs. 6, 7). Both methods give practically the same results as shown here by the median and extreme values (quantiles 10% and 90%) of the firing rates (Fig. 8A) and the latencies (Fig. 8B).

#### Dose-response curves

First, at any dose *C*<1 log ng, the median firing rate is lower in ORNs than in PNs; this results in a higher sensitivity in PNs with a shift of the whole median *C*-*F* curve towards lower doses. The two median *C*-*F* curves based on the median values of the parameters having similar slopes in ORNs (*n* = 0.81) and PNs (*n* = 0.79) the shift is almost constant at all doses; 2.7 log units separate their *C*_1/2_, indicating a∼500-fold (10^2.7^) increase in sensitivity from ORNs to PNs (Fig. 8A). This shift is apparent also in the distributions of the doses *C*_0_ (Fig. 8E) and *C*_1/2_ (Fig. 8F), whose means are shifted in PNs to the left of ORNs. The separations between ORN and PN distributions are maximal at certain doses (Fig. 8E, F); the largest separation is for *C*_1/2_ with 98% of PNs but only 2% of ORNs having a *C*_1/2_ smaller than 0 log ng (denoted *C*_1/2Δ_, dashed black curve in Fig. 8F). Second, their response latencies at the same dose are also very different, the median latency being ∼90 ms shorter in PNs than in ORNs, and the typical PN line being shifted by ∼5 log units to the left of the ORN line. In particular, minimum latencies *L*_m_ in ORNs are 58% longer than in PNs (*t* test, *p*<10^−4^). The latencies at the dose *C*_1/2Δ_ determined above are twice shorter in PNs than in ORNs; thus, at this dose, only 22% of ORNs but 90% of PNs have latencies shorter than 111 ms.

#### Transfer function

The median transfer function for firing rate is close to a branch of hyperbola (Fig. 8C) that can be approximated as a piecewise linear function with two segments. The first line is steep, the PN firing rate jumping from 0 to 40 AP/s for a small increase of the ORN rate from 0 to ∼2 AP/s. In this range each AP/s in ORNs evokes>20 AP/s in PNs. The second line (for>25 AP/s in ORNs and>60 AP/s in PNs) is flat; each AP/s in ORNs in this range evokes only ∼0.03 AP/s in PNs. The median transfer function for latency is linear (Fig. 8D). So, apart from the irreducible minimum latency *L*_PN_ ≈43 ms for *L*_ORN_ <128 ms, the ORN-to-PN transformation typically reduces the ORN latency by a factor of 1/0.65 = 1.55.

### Sensitivity and speed are not correlated

So far only univariate distributions have been considered since the 11 properties that describe the *C-F* curves (fitted parameters *F*_M_, *C*_1/2_, *n* and derived characteristics *C*_0_, *C*_s_, *ΔC*) and *C-L* curves (parameters *L*_0_, *λ*, *L*_m_ and characteristics *L*_M_, *ΔL*) were analyzed separately. This analysis must now be completed by examining the links between them. For this purpose bivariate plots and their associated correlations were studied. S5 Table assembles the coefficients of correlations and significance levels of the (11^2^−11)/2 = 55 non-trivial pairs of properties in ORNs (top) and in PNs (bottom). It shows that 19 pairs (35%) are significantly correlated at level 1% in ORNs and also 19 in PNs, indicating that the overall correlative structure is similar in both populations. However, because characteristics are derived from parameters (for example Δ*C* depends on *F*_M_ and *n*, see eq. 7), correlations between characteristics are expected to be more frequent and less informative than the correlations between parameters. Indeed, of the (6^2^−6)/2 = 15 pairs of parameters only 3 (20%) are significantly correlated in ORNs and the same number in PNs. Therefore, in the following, priority is given to the parameters over the characteristics.

Another distinction is between two main types of pairs – those associating properties of the same variable, firing rate or latency, that provide information on the level of redundancy of properties, and those crossing the two variables that provide information on the link between them.

In the first type, of the 3 pairs of *F*-parameters (*F*_M_-*C*_1/2_, *C*_1/2_-*n* and *n*-*F*_M_) none is significantly correlated (*p*>0.01) in ORNs (S2A-C Figure) and a single one in PNs (*C*_1/2_-*n*, S3B Figure), indicating that the most sensitive PNs (with small *C*_1/2_) tend to have a steeper slope *n*. Similarly, of the 3 pairs of *L*-parameters a single one is uncorrelated in both ORNs and PNs, suggesting that *L*-parameters are more correlated to one another than *F*-parameters. The uncorrelated pair (*λ*-*L*_m_, S2E, S3E Figures) indicates that neurons with longer latencies at *C* = 0 tend to have steeper slopes *λ* and longer minimum latencies *L*_m_.

As for the two-variable type, among the 9 pairs between the 3 *F*- and 3 *L*- parameters, a single one is significantly correlated but it is not the same in ORNs (*C*_1/2_-*L*_0_, S2J Figure) and in PNs (*C*_0_-*L*_m,_ S3P Figure). Although the correlation *C*_1/2_-*L*_0_ suggests that fast ORNs (small latency *L*_0_ at *C* = 0) have high affinity (small *C*_1/2_), it is not confirmed by direct comparison as threshold *C*_0_ and minimum latency *L*_m_ in each ORN are not correlated (S2P Figure), showing that the ORNs with the lowest thresholds are usually not the fastest. As the reverse situation holds for PNs, this lack of consistency and the low proportion of significant correlations between *F-* and *L*-parameters (11%), and still more between *F*- and *L*-properties (10% for ORNs, 7% for PNs), support the overall independence of sensitivity and speed in ORNs and PNs.

### Heterogeneity across neurons is the main source of variability

The firing rates and latencies are variable across ORNs (and PNs) stimulated at the same dose (Figs. 4, 5). As shown below variability plays an essential role in the ORN-to-PN signal transformation which calls for a proper understanding of its sources and structure. First, variability arises from experimental and biological sources. Experimental variability in ORNs results from uncertainties on the dose and the delivery time from the stimulating device, and from the relative geometry of the airflow and the recorded sensillum. It must be reduced but can never be eliminated. Biological variability is more fundamental because it is an intrinsic property of the investigated system. It can be known only by subtracting the experimental variability from the overall observed variability. Second, variability arises from irregularities within single units and heterogeneities across units, where units can be neurons or pheromone stimuli. This distinction is important. The term ‘irregularity’ is used consistently throughout the paper to indicate the variability in firing rate or latency of the same neuron, or the variability on dose or delivery time of the same stimulus, following repeated stimulations with the same cartridge. By extension it indicates also the variability of spontaneous firing of the same neuron over time. Similarly the term ‘heterogeneity’ indicates the variability in response of different neurons, or the variability on dose and delivery time for repeated stimulations with identically prepared cartridges.

Systematic measurements (see Materials and Methods) showed that the biological component accounts for ∼90% of total variability. Biological variability results more from heterogeneity across ORNs (∼95% for *F*, ∼55% for *L*) than from irregularity within ORNs. These observations support a relatively simple interpretation, that the observed variability of ORN responses reflects primarily across-neuron heterogeneity. When compared to ORNs, the irregularity within PNs was smaller for firing rate (70%) and latency (80%), and the heterogeneity across PNs was equal for firing rate (Fig. 5E, superimposed blue and red lines) and larger for latency (140%, Fig. 5F, lines with different slopes).

Of special interest from a population coding point of view are the most active and the fastest neurons. To estimate the effect of the heterogeneity of ORNs and PNs and reconstruct their full range of variability, we selected the 10% most extreme values at both ends of the distributions of the parameters describing the dose-response curves (staircases in Figs. 6, 7). Fig. 8A shows that the *F*/*F*_M_ curves of the 10% most efficient ORNs (derived from leftmost blue dashed curve) and the typical PN (derived from red solid line) are relatively close. This observation is also true for the *C*-*L* curves (Fig. 8B). Thus, the 10% most efficient PNs are likely triggered by a small fraction of ORNs. The transfer functions provide useful complements. The function of the 10% most (respectively least) efficient neurons (Fig. 8C, dashed and dash-dot lines) is less (respectively more) curved than the average function (solid line). The function of the 10% fastest neurons is a steep line that decreases to shorter latencies (Fig. 8D) than the median function.

### The increased performance of PNs cannot be explained by ORN-to-PN convergence alone

**Figure 9:**
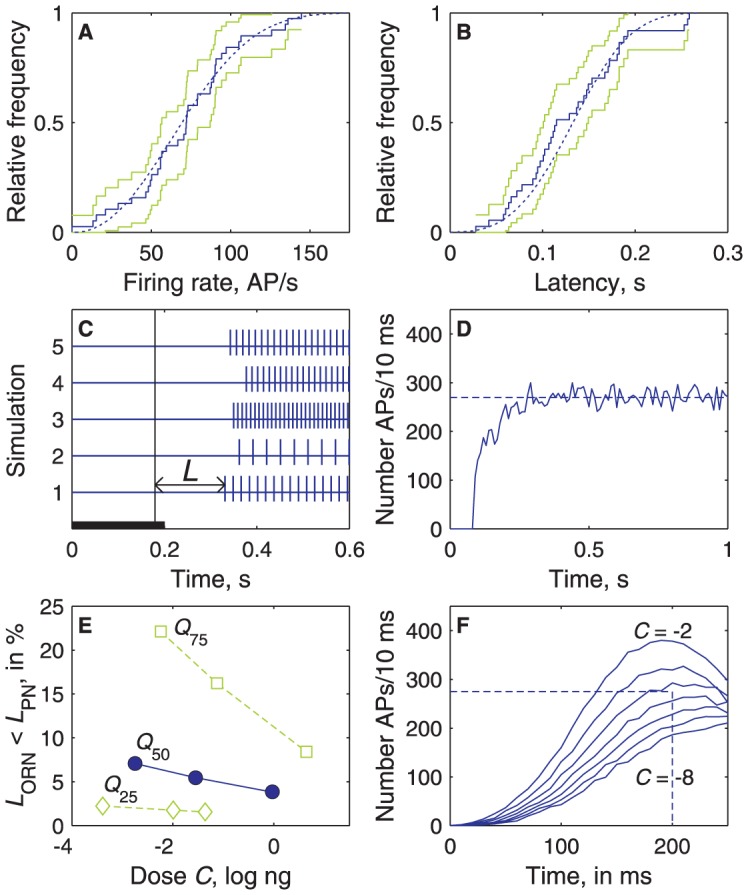
Model of a large ORN population converging on a small PN population. (**A, B**) Example at dose *C* = 1 log ng of cumulated distributions of modelled firing rates (A) and latencies (B) of ORNs and comparison with experimental data. Distributions of experimental values (*N* = 32, same as in Fig. 5A, B) shown as staircase graphs (solid line) with 95% confidence intervals (green lines). Distributions of modelled *F* and *L* shown as smooth curves (in blue) based on *N* = 5 000 drawings. Firing rates in the model obey eq. 4 and latencies eq. 8 with parameters *L*_0_, ln(*λ*), *L*_m_, *F*_M_, *C*_1/2_, ln(*n*) drawn from a multinormal distribution with their observed means, SDs and correlations (S5 Table). The modelled and experimental distributions are not significantly different (Kolmogorov–Smirnov tests at level 1%). (**C**) Examples of 5 simulated ORN responses with interspike interval 1/*F* and latency *L* at *C* = 1 log ng for 5 parameter drawings; 7000 such responses are summated to simulate the whole ORN population). (**D**) Simulated spontaneous activity of the whole ORN population (based on the lognormal distribution with μ = 1.23 and σ = 0.71 shown in Fig. 3E); the firing rate fluctuates around a stationary value ∼270 AP/10 ms. (**E**) Proportion of ORNs that respond with a shorter latency than the typical PN (with all 6 parameters equal to their median values; solid line), than a slow and insensitive PN (parameters equal to their 75% quantiles, dotted), and than a fast and sensitive PN (parameters equal to their 25% quantiles; dashed) at doses *C*_0_, *C*_1/2_ and *C*_s_. (**F**) PSTH of the total number of spikes fired per 10 ms at doses from −8 to −2 log ng by a simulated population of 7000 NROs. The summated firing rate close to detection threshold (dotted line, 275 APs per 10 ms, see text) is reached for *C*≈−4.5 log ng at time ∼200 ms.

Not all ORNs in the population contribute equally to the PN response. The major contribution comes from the ORNs whose latency is shorter or equal to the PN latency, since no PN can respond faster than its presynaptic ORNs. In order to determine the fraction of contributing ORNs we relied on a model based on the *C*-*F* and *C*-*L* curves and the distributions of their parameters established above (see last section “Model of the signal delivered by the ORN population” in Materials and Methods). The model predicts the firing rates and latencies observed in the ORN population (Fig. 9A, B) and allows us to simulate the spike trains fired by this population when stimulated (Fig. 9C) and in the absence of stimulation (Fig. 9D). From these simulations, we calculated at any dose *C* the proportion of ORNs that respond with a given latency *L*_1_ or shorter. This proportion, as shown in Fig. 9E when *L*_1_ is the latency of the typical PN reconstructed from the median values of the fitted parameters, decreases with the dose and only 5±2% of the ORNs are enough to activate the typical PN. The proportion is greater (16±7%) for the slow PNs and smaller (2±0.3%) for the fast ones.

The response kinetics were obtained from the model by simulating the total firing of the heterogeneous population of ∼7000 Z7-12:Ac ORNs knowing the statistical distributions of their experimental *C*-*L* and *C*-*F* curves. At all doses, the simulated PSTH shows that the instantaneous firing rate of the ORN population increases and reaches a maximum ∼200 ms after the stimulation onset (Fig. 9F). The initial growth results from the gradual recruitment of new ORNs. Then, the pheromone dose at threshold can be determined. The ORN population spontaneously fires ∼27 000± SD √27000 AP/s (Fig. 9D). To be detected by PNs, the firing rate must be greater than 27000+*r*√27000, where *r* is the signal-to-noise ratio. In another moth species, *Bombyx mori*, Kaissling [25] showed that, in the range of pheromone loads eliciting a behavioral response in 40% to 80% of male moths (0.01 to 0.1 ng per cartridge in his experimental conditions), *r* varies from 3 to 31. Assuming that the same range of ratios applies to *Agrotis*, the ORN population must fire from 27 500 AP/s (for *r* = 3) to 32 000 AP/s (for *r* = 31). Fig. 9F shows that the lowest stimulus doses evoking 275–320 AP per 10 ms are in the range −4.6 to −3.1 log ng. At this dose the typical PN fires only 0.3–3.3 AP/s.

## Discussion

In this study, our goal was to interpret the input-output transformations taking place in an identified glomerulus stimulated with a well-defined odorant. We focused on how functionally homogeneous populations of first- (ORNs) and second- (PNs) order neurons with heterogeneous characteristics encode the odorant concentration in their firing rate and in the latency of their first spike. To this end we chose a glomerulus specialized in the processing of the main component of a sex pheromone. This glomerulus with its associated ORNs and PNs is essentially similar to the other glomeruli in the antennal lobe so that the conclusions drawn from its study are of general applicability. However, it presents decisive advantages for the intended investigations because its ORNs are selectively activated by the pheromone component, do not project to other glomeruli, are the most numerous in the antenna and present the largest ORN-to-PN convergence ratio of all glomeruli.

### Intensity coding by firing rate and latency

Single ORNs and PNs encode the pheromone concentration in the same way, their dose-firing rate curves being well fitted to Hill functions – a classical fit for ORNs [25], [26]. The maximum firing rate *F*_M_ of ORNs is usually much larger in insects (100–300 AP/s; e.g. [6], [27] with an exception <60 AP/s, [28]) than in vertebrates (13–50 AP/s, [26]). The same remark holds for PNs in insects (170–250 AP/s; [6], this work) and the analogous mitral cells in vertebrates (∼20 AP/s in frog; [29]). Also, the dynamic range *ΔC* is much wider in pheromone-sensitive ORNs than in vertebrate generalist ORNs (usually <2 log units, [26]). Because, for a given Hill coefficient, *ΔC* depends on *F*_M_, it is tempting to speculate that the reason why the firing rate of pheromone-responsive neurons is so high, despite its large energetic cost, comes from the importance of detecting pheromones at very low concentration. The high firing rate would then be the price to pay to have a low threshold and a wide dynamic range.

The median latencies *L*_M_ at threshold in *A. ipsilon* in ORNs (164 ms) and PNs (107 ms) are consistent with those in non-pheromonal honeybee PNs (∼125–150 ms; [12]), and much shorter than those in frog ORNs (0.7–1.9 s; [25]). A possible interpretation of the faster response of insect ORNs is that insect ORs were proposed to be ionotropic [30], [31] whereas vertebrates ORs are metabotropic [32]. These observations raise the question of whether latency actually contributes to encode pheromone concentration because the stimulus onset defining time zero is not known by the brain. However, it has been shown that relative latencies, *i.e.* patterns of spikes, can be used as a replacement of stimulus onset [11], [15], [33]. This applies when successive stimuli are well separated (as in pheromone plumes where molecules remain grouped in clumps and filaments separated by clean air [34]) and even when they are not, for example in an ensemble of mitral cells [20]. Moreover, latency coding is consistent with temporal coding mechanisms in which the precise timing of action potentials carries information on the stimulus [35]. It is now recognized that temporal coding is widely used in all sensory systems, whether olfactory [36], tactile [37], [38], auditory [39], or visual [40].

### ORN-to-PN transformations

The overall transformations of the olfactory code from ORNs to PNs can be determined in two different ways, either directly at the population level from their pooled responses at a given dose (Figs. 4, 5) or indirectly from responses of individual neurons across doses (Figs. 6, 7). The two approaches are consistent as the same transformations were found in both (Fig. 8A, B) strengthening a posteriori the more complex but more informative single-cell analyses.

#### Transformation of firing rate

The transformation of firing rate from ORNs to PNs is characterized by a shift toward low doses of the dose-firing rate curve of PNs with respect to that of ORNs and by different slopes of a single transfer function relating the frequencies of ORNs and PNs at the same doses. Both effects manifest signal amplification from ORNs to PNs at low doses. The *C*-*F* representation makes clear that PNs have a lower threshold than ORNs and displays other effects like changes in slope and maximum firing rate (Fig. 8A). The *F*-*F* representation emphasizes the amplification of the weakly active ORNs over the strongly active ones (Fig. 8C).

The same dual description applies to the transformation from the number of activated ORs to the receptor potential in ORNs, as both variables are sigmoid functions of the dose shifted with respect to one another by ∼2.2 log units [41]. These transformations at the cellular and network levels add their effects and move the ED50 of PNs by ∼5 log units with respect to ORs.

Does the transfer function apply also in the absence of stimuli? When the antenna is sectioned, the spontaneous activity of PNs (12 AP/s) being reduced by ∼70% appears as the sum of a major component induced by ORNs (70%, *i.e.* 8 AP/s) and a minor component (30%) intrinsic to the MGC. This result indicates that, contrary to the locust AL [42], spontaneous activity in the moth MGC is not inherited entirely from the ORNs. Moreover, the amplification at rest of spontaneous activity from 3.8 AP/s in ORNs to 8 AP/s in PNs (Fig. 3E) is lower than that expected from the median transfer function (Fig. 8C) and suggests the existence of mechanisms preventing noise amplification.

#### Transformation of latency

Similar representations apply to the transformation of latencies. The *C*-*L* representation shows that PNs have shorter latencies than ORNs at any dose (Fig. 8B) as observed in the generalist glomeruli of the fly using PSTH [6], [43]. The *L*-*L* representation reveals that the PN latency is a linear function of the ORN latency, whatever the dose and the speed of the neurons (Fig. 8D). This indicates that the PN latency is the sum of 2 components. The first component (ordinates at the origin of the transfer functions, range ∼20-80 ms) is the time due to pheromone diffusion in the antenna, transduction, axonal conduction and synaptic transmission. The second component (slopes of the transfer functions) is proportional to the ORN latency; the constant of proportionality is ∼0.7 for median neurons, so PNs are ∼40% times faster than ORNs.

#### Structural and random components of variability

Differences across neurons appear in the diversity of observed dose-response curves. The diversity in moth pheromonal ORNs is consistent with the large variability in threshold, dynamic range and maximum current found in mouse ORNs expressing the same OR [3]. It is smaller than the dispersion of thresholds and ED50s observed in frog and rat generalist ORNs expressing different ORs [25], [26]. However, the overall variability observed results from several sources. Two main types of variability were distinguished.

The first type is the irregularity of spontaneous and evoked activity within individual cells. It can be considered as noise. Spontaneous activity fundamentally limits the recognition of ORN (and PN) responses by the experimenter at low doses. Once a response is recognized, the precision with which it is known is limited by the irregularity of the stimulus and the transduction process. However, measurements showed that this noisy component accounts for at most 40% of the total variability on latency and less than 20% on firing rate (see section “Measurement of the component of variability” in Materials and Methods).

The second type and major (60-80%) source of variability is the heterogeneity of ORNs in the population. It is a structural property of the system that results from variations in the level of the various components (OR, channels etc.) involved in transduction [44] or in membrane areas of various parts of the sensilla [45]. The moth single-glomerulus ORN-to-PN transfer functions (Fig. 8C) are similar to those observed in the fruit fly [6], [46]. For odorants stimulating a single glomerulus, without lateral input coming from other glomeruli, the fly *F*-*F* functions [7] correspond to those found here. The heterogeneity across neuron pairs in the moth and across glomeruli in the fly [6], [7] is visible in the angle between the initial (∼vertical) and final (∼horizontal) branches of the *F*-*F* curves, reflecting diverse values of the ORN input *F*_1/2_ that drives half-maximum PN response. The *F-F* transfer function (eq. 9) shows that this angle gets closer to 90° when the difference *ΔC*_PR_  =  *C*_P_ – *C*_R_ between the *C*_1/2_ of the PNs and ORNs or when the ratio *n*_P_/*n*_R_ of their Hill coefficients increases. The main difference between the parameters of the Hill functions fitted to the empirical *F-F* curves in the fly and the moth concerns the ORN input *F*_1/2_ which is 12–45 AP/s in the fly [7] and 1.50 AP/s for the moth median *F*-*F* curve. The 10 to 30-times larger input in the fly glomeruli than in the moth cumulus can be readily interpreted by the smaller convergence ratio (and therefore *ΔC*_PR_) in the fly.

### Role of convergence and heterogeneity

Coding properties at the system level depend on the many-to-one ORN-to-PN convergence and individual differences between neurons in the ORN and PN populations.

#### The high affinity of PNs can be explained by convergence

The increased performance of PNs in sensitivity, as measured by the threshold or ED50, can be explained by the convergence of many ORNs on single PNs. The heterogeneity of the ORN population does not play any essential role with respect to affinity because a similar amplification is obtained when considering all ORNs as Poisson neurons with the same firing rate [4]. The number of Z7-12:Ac ORNs (∼7000) is much larger than the number of their postsynaptic PNs (∼10–70 according to ratios estimated in other moth species), so that each PN can monitor the activity of many ORNs. This high convergence ratio can account for the ORN-to-PN transformation in firing rate and consequently the improved sensitivity of PNs [4]. It is also consistent with the suggestion that spontaneous activity from ORNs may be used to maintain PNs near the spiking threshold and increase PN sensitivity [37]. With each PN monitoring the summated activity of a large number *N* of ORNs, a multiplication by √*N* of the signal-to-noise ratio can be obtained, which explains that PNs start to respond at doses for which only a small fraction of ORNs have started to respond conspicuously; for example at −1.9 log ng, 17% of the ORNs can trigger 85% of the PNs (Fig. 8E).

#### The high speed of PNs cannot be explained by convergence alone

The shorter latencies of PNs with respect to ORNs are more delicate to interpret than their higher frequencies at the same dose as they are subject to several pitfalls. PN short latencies may seem paradoxical because it suggests that PNs start to respond before ORNs. In fact, it is not necessary that a large proportion of ORNs responds to trigger their postsynaptic PN, a few fast ORNs might be enough. At high doses (*C*>0), ORNs almost as fast as the fastest PNs can be directly observed (Fig. 4H). At lower doses, that number decreases and recording from these fast ORNs becomes an increasingly rare event (Fig. 4F) lost in the noisy background of the spontaneous activity. Because of spontaneous bursts, the minimum response recognizable in ORNs is ∼15 AP/s. Therefore no ORNs, even the most sensitive ones, can display significant responses at doses lower than *C*≈−1 log ng. This limitation implies that no direct comparison between experimentally measured responses in ORNs and PNs can be done at doses *C*<−1 (Fig. 4) and illustrates the fact that the limitations met by the experimenter and the system are not the same. Thus, at low doses, ORN latencies shorter than PN latencies could be recorded only in ORNs having both very fast responses and no spontaneous activity.

In contrast to their high affinity, the short latency of PNs cannot be explained by convergence alone. If the latency of all ORNs was the same, the PNs would have at best the same latency as the ORNs. Therefore, convergence and ORN heterogeneity are essential to account for the faster response time of PNs. Our convergence model suggests that at least ∼275 APs per 10 ms (Fig. 9F) are needed to activate the typical PN with parameters equal to the median values. This is consistent with the ∼200 ORNs eliciting a behavioral response in the silk moth [47] (assuming that an ORN cannot fire more than one spike in 10 ms). So, PN speed implies a relatively large ORN population and is thus an example of population coding [27]. However, this form of population coding based on the reaction to only a few active neurons in a population should be distinguished from other forms based on averaging activity over many neurons, like in the case of firing rate.

Can other explanations of the shorter latency of PNs with respect to ORNs be proposed? PN responsiveness might depend on its past stimulation history based, for example, on the mechanism of anticipated synchronization demonstrated in a neural network similar to the cumulus network [48]. However, no evidence for history dependence of this kind has been found as short latencies appear even in a “naïve” preparation and do not shorten with repeated stimulations at the same dose. Moreover, anticipatory mechanisms in PNs do not seem promising because of the highly unpredictable nature of pheromone plumes resulting from atmospheric turbulence.

Another objection is that the convergence of many ORNs on single PNs should average out the variability of ORNs. With a convergence ratio *N*:1 (*N* ≈ 300 ORNs per PN) the decrease in variability of PNs with respect to ORNs should be √*N* instead of the modest decrease observed in irregularity (70–80%) and the stability or even increase in heterogeneity (100–140%). A possible interpretation is that the intrinsic irregularity and heterogeneity of PNs are high because of cellular differences and variations in the number and strength of their connections [49]. The PN diversity is reminiscent of that reported in cortical neurons (e.g. [50]) and in sister cells innervating the same glomerulus in the fly [51] and the mouse [52] (but see [53]). This response heterogeneity, found in different organisms, has been shown to improve the population codes [52], [54], [55].

### Efficient coding

The ORN-to-PN transformations, which entail stronger and faster responses, considerably differ quantitatively, the transformations at low doses being linear in latency and highly nonlinear in firing rate. These effects may be partly interpreted within the “efficient coding hypothesis” [56], [57] stating that the sensory neurons, including the moth pheromonal ORNs [58], are adapted, through both evolutionary and developmental processes, to the statistics of their natural stimulus.

The effect of the nonlinear ORN-to-PN firing rate transfer function is to give more weight to the low doses than to the high doses. The typical PN reaches saturation at a relatively low dose (*C*_S_ ≈ 0 log ng) with respect to ORNs (Fig. 8A) and so cannot discriminate doses above 0 log ng. Such a transformation is reasonable from an efficient coding point of view because the high pheromone concentrations found within filaments far from the source [59] are rare and not informative for localizing the source. This nonlinear transformation is reminiscent of logarithmic companders for coding waveforms with a wide dynamic range, such as voice, on a finite number of levels, favoring the most frequent signals at the expense of the least frequent ones [60]. The same interpretation can be applied here to the coding of pheromone concentration (e.g. a lognormal distribution of concentrations in nature will be encoded more uniformly by PNs). In the fly this type of transformation was interpreted as favoring the qualitative discrimination of odorants. At any dose, odorants evoke a wide range of firing rates in ORNs [61] and PNs but their distribution is more uniform in PNs [6]; the hyperbolic transfer function explains this histogram equalization. Thus, the nonlinear ORN-to-PN transformation can be interpreted as favoring qualitative discrimination of odorants in the generalist pathway and detection at low doses in the pheromonal pathway.

In contrast, the linear latency transfer function preserves the type of statistical distribution between ORN and PN latencies (e.g. a Gaussian remains Gaussian) and so cannot perform histogram equalization. A possible advantage of this linearity is to make the coding of pheromone identity concentration-invariant [11]. In *A. ipsilon*, although the ratio of concentrations of one of the minor components to the major component is 4, linear latency functions with the same slope will introduce the same constant delay between the specialized ORNs responding to the components whatever the blend concentration. Any change of the ratio will produce a detectable change in the delay, signaling an inadequate pheromone blend.

In this view, the ORN-to-PN convergence may be more important for fast detection of the signal than for precise determination of its intensity. The sensitive response of the PNs to the fastest ORNs and their saturation at relatively low doses make the PN output more stable to dose variations than the ORN output and favor information on the temporal structure of the plume over its concentration fluctuations. The cumulus appears to obey the same principles as the ordinary glomeruli with a notable difference. Because of a higher ORN-to-PN convergence ratio, the response of PNs in the cumulus is presumably more sensitive and faster than in ordinary glomeruli. Temporal discrimination, a constraint in ordinary glomeruli, becomes apparently a major issue in the MGC.

## Materials and Methods

### Insects

*Agrotis ipsilon* (Lepidoptera: Noctuidae) were reared on an artificial diet until pupation [62]. Adults were fed with a 20% sucrose solution. All experiments were performed on sexually mature virgin males 5 days after emergence.

### Stimulation

A constant airflow (24 ml/s) was blown over the antenna through a mixing tube. It resulted from mixing a constant airflow (17 ml/s) with an alternating flow (7 ml/s) of clean air between stimulations or odorized air during stimulations. Stimuli were delivered by means of a device (Syntech, Kirchzarten, Germany) blowing air during 200 ms through a Pasteur pipette containing the pheromone-impregnated filter paper and inserted into the mixing tube. Successive stimulations were separated by intervals of at least 60 s in ORNs and 30 s in PNs; these intervals are sufficient for complete return to spontaneous activity (Fig. 2E), except with the highest doses tested (3 and 4 log ng for ORNs and 1 log ng for PNs) for which longer intervals were used. In electrophysiological experiments, the main pheromone component of *A. ipsilon*, (Z)-7-dodecen-1-yl acetate (Z7-12:Ac) was used at different loads *M* (1 pg to 10 µg). In calcium imaging experiments also the two minor pheromone components (Z)-9-tetradecen-1-yl acetate (Z9-14:Ac), and (Z)-11-hexadecen-1-yl acetate (Z11-16:Ac) [63] were used for stimulation at a single load (10 ng). Doses denoted *C* were expressed as the decimal logarithm of loads in ng.

### Calcium imaging

All 3 components of the pheromonal blend [63] were tested (Fig. 1). Recordings were performed as described in [64]. Briefly, Calcium Green 2-AM was bath-applied for at least 1 hour. A TILL Photonics imaging system (Martinsried, Germany) was coupled to an epifluorescent microscope (Olympus BX-51WI) equipped with a 10x (NA 0.3) water immersion objective. Signals were recorded using a 640×480-pixel, 12-bit monochrome CCD camera (TILL Imago).

### Electrophysiology

Only the major component (Z7-12:Ac) was tested. Recordings from ORNs were performed as described in [62]. Briefly, the glass recording electrode was brought into contact with a cut sensillum and the reference electrode was inserted in an adjacent antennal segment. Great care was taken to cut the sensilla at the same short distance from their tip. Each ORN was recorded for 10 s before and 40 s after the onset of each stimulus at 6 doses from -1 to 4 log ng. Only recordings with spikes clearly attributable to a single ORN were kept for further analysis. PNs were recorded from the cumulus area with two different techniques. Extracellular recordings (_E_PN) were performed with glass electrodes of 5 MΩ resistance as described previously [65]. The electrical activity of one or several neurons was recorded. Spike-sorting was performed with the R-package SpikeOMatic [66]. All _E_PNs were stimulated twice at five doses from −3 to 1 log ng. For each stimulus, 12 s of post-stimulus activity were recorded. Intracellular recordings (_I_PN) were performed with a glass electrode of 150–200 MΩ resistance filled with Lucifer Yellow or neurobiotin as described in [67]. _I_PNs were tested at four doses, −2, −1, 0 and 1 log ng and recorded for 3 s after the onset of stimulation. The brain was then dissected, histologically treated and scanned in a confocal microscope as a wholemount. All _I_PNs kept for analysis shared the same physiological and (when available) morphological characteristics of PNs. Only a few LNs were impaled in our recording conditions and they never showed phasic response patterns. The distributions of the response frequencies and latencies of _I_PN and _E_PN were compared by Kolmogorov-Smirnov tests. No significant difference was found, so the two samples were pooled.

### Response frequencies and latencies

The time varying spike rate function *f*(*t*) was estimated by using the kernel method [68]. The spike trains of ORNs and PNs were convoluted with a Gaussian function of SD 50 ms. The raw response firing rate *F*_raw_ was defined as the height of the peak of *f*(*t*) (S1B, D Figure). In PNs with triphasic responses (excitation-inhibition-excitation) *F*_raw_ was determined on the first peak. Height *F*_raw_ was compared to the bumps of *f*(*t*) during spontaneous activity in the same neuron. The number of bumps of height *f* ≥ *F*_raw_ was counted; when it was less than 5% of the total number of spontaneous bumps, response *F*_raw_ was considered as significant.

Response time *T* is the time elapsed from the opening of the electric valve to the first spike of the response. For ORNs (S1A Figure), this spike is defined without ambiguity because of their low spontaneous activity. For PNs (S1C Figure), a few ambiguous cases due to spontaneous activity were resolved visually (the correction is conservative as it always increases *T*). Response latency *L* was defined as the difference between *T* and the mean transport time *T*_t_ (180 ms), *L* = *T* – *T*_t_. As *T*_t_ follows a Gaussian distribution with σ≈13 ms (80% values are in the range 170-210 ms); a minor drawback of this definition is that at high doses the shortest latencies of PNs (not ORNs) can become negative if *T*_t_ is short.

Control responses (Fig. 4A, B) resulting from puffs of the solvent (hexane) were recorded in ORNs (15± SD 10 AP/s) and from puffs of hexane and non-odorized air in PNs (59± SD 28 AP/s; the distributions for hexane and pure air were pooled as they were not significantly different, *p*-value 0.72). To obtain the pure olfactory component *F* the average *F*_c_ of the control responses in each neuron was subtracted from the responses *F*_raw_ measured in the same neuron, *F* =  *F*_raw_ – *F*_c_. Rate *F*_raw_ is shown only in Fig. 4A, B; all other figures use the pure component *F*.

### Measurement and modelling of the components of variability

#### Stimulus (S4A-D Figure)

In order to determine the influence of stimulus variability, measurements were done with a photoionization detector (Aurora Scientific Inc., Canada) and α-pinene as stimulus because Z7-12:Ac cannot be ionized. Means and SDs of PID signals were measured in amplitude and onset time in 10 series of 14 repeated stimuli, within series using the same stimulus cartridge (pipette, filter paper and load; irregularity) and across series (with different cartridges; heterogeneity). For onset time, the SDs were constant, with heterogeneity (SD  = 13.3 ms) 3 times larger than irregularity (4.43 ms). For amplitude, the SDs were found proportional to the mean amplitude, preserving the constancy of the coefficient of variation (CV  =  SD/mean) at two loads of α-pinene (4 and 5 log ng), with heterogeneity (CV  = 0.195) 4 times larger than irregularity (0.05).

#### ORN responses (S4E-H Figure)

Irregularity and heterogeneity in firing rate and latency of an ORN sample were measured in similar experiments (10 series of 14 repetitions). *F* and *L* at a given dose are normally distributed; their means and SDs increase with the dose (Fig. 5E, F), whereas their CVs remain constant (except for *F* at *C*≥1 for ORNs and *C*≥0 for PNs where SDs become constant, Fig. 5E). For firing rate, heterogeneity (CV  = 0.60) is larger than irregularity (0.16). For latency, heterogeneity (0.43) is larger than irregularity (0.34).

#### Analysis

The observed irregularity of single ORN responses (variance σ_Ti_^2^, ‘T’ for total) results from their unknown intrinsic biological irregularity (variance σ_Bi_^2^, ‘B’ for biological) and stimulus irregularity (variance σ_Si_^2^). These two components are independent, so
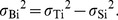
(1)


The ratios σ_Bi_^2^/σ_Ti_^2^ show that 90–95% of ORN irregularities in firing rate and latency are accounted for by intrinsic properties.

Similarly, the observed response heterogeneity σ_Th_^2^ is the sum of three components − stimulus heterogeneity σ_Sh_^2^, intrinsic irregularity within single ORNs σ_Bi_^2^, as determined above, and unknown intrinsic heterogeneity σ_Bh_^2^, so
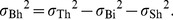
(2)


For firing rate, the contributions of σ_Bh_^2^ to total variance σ_Th_^2^ (∼85%) and to total biological variance σ_Bi_^2^ + σ_Bh_^2^ (∼95%) remain constant. For latency, the contribution of σ_Bh_^2^ to total variance (∼30%) tends to decline at high doses where it becomes small whereas its contribution to biological variance (∼60%) is almost constant.

### Fitting of response variables to dose

For each set of recordings from a neuron, empirical functions were fitted to the experimental points in the dose-response plots *C*-*F* (Fig. 6) and *C*-*L* (Fig. 7).

#### Firing rate

The frequencies *F* at different loads *M* of any neuron (ORN or PN) can be described by a Hill function of *M*

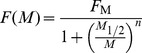
(3)with *F*_M_, asymptotic maximum firing rate at high loads, *M*_1/2_ load at half-maximum response *F*_M_ */*2, and *n* Hill coefficient. Using dose *C* =  log_10_ *M* as the variable, function (3) becomes
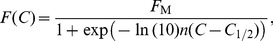
(4)where *C*_1/2_ =  log_10_ *M*_1/2_. The parameters of the function (4) fitted to data were estimated by an iterative nonlinear least square method (Fig. 6A). Three characteristics were derived from the fitted parameters. The response threshold *C*_0_ of ORNs and PNs was determined as the dose at which *F* rises above *F*_0_ = 5 AP/s. The saturation dose, *C*_S_, was determined as the dose at which *F* reaches *F*_M_ *– F*_0_. The dynamic range was defined as the difference between these two values
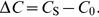
(5)Δ*C* is a dimensionless quantity that expresses in log units the ratio of loads at saturation and at threshold; for example a dynamic range of 2.5 log units (median value for PNs) means that the load at saturation is 10^2.5^ = 316 times larger than the load at threshold.

It can be shown that 
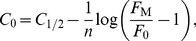
(6)

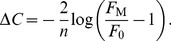
(7)


Eq. 7 shows that *n* and Δ*C* are inversely related. When referring to both parameters and characteristics, we used the term ‘properties’. The 6 firing rate properties are given in S3 Table.

#### Latency

The latencies *L* of each ORN and PN were fitted to a linearly decreasing function of *C*:
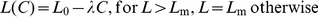
(8)with two parameters: latency *L*_0_ at dose *C* = 0, and slope *λ*. For each neuron, *L*_0_, and *λ* were determined by a linear least-square regression and minimum latency *L*_m_ was determined as its shortest measured latency (Fig. 7A). Two characteristics were derived from these parameters, the maximum latency *L*_M_ and the range of latencies *ΔL*. *L*_M_ was calculated from eq. 8 as *L*_M_ = *L*_0_−*λC*_0_, with threshold *C*_0_ determined from eq. 6. *ΔL* was defined as *ΔL = L*_M_ – *L*_m_. The 5 latency properties are given in S4 Table.

### Transfer functions for firing rate and latency

From eq. 4, the firing rate *F*_P_ of a PN at any dose *C* can be derived as a function of the ORN firing rate *F*_R_ at the same dose. Denoting (*F*_RM_, *C*_R1/2_, *n*_R_) the ORN parameters and (*F*_PM_, *C*_P1/2_, *n*_P_) the PN parameters, the transfer function for firing rate is
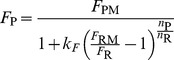
(9)where 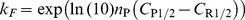
(10)is a constant. Except for the −1 term in the denominator, function (9) has the same form as Hill function (4).

Eq. 9 can be derived from the ORN and PN Hill functions. However, the reverse is not true because the absolute positions of the firing rate curves along the dose axis (*C*_R1/2_ and *C*_P1/2_) are lost in the transfer function. Thus, a pair of dose-firing rate curves contains more information (6 parameters) than the corresponding transfer function (5 parameters).

Similarly, the latency *L*_P_ of a PN at any dose *C* can be expressed as a function of the ORN latency *L*_R_ at the same dose. Denoting (*L*_R0_, *λ*_R_) the ORN parameters and (*L*_P0_, *λ*_P_, *L*_Pm_) the PN parameters, it can be shown from eq. 8 that 
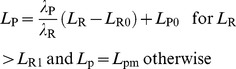
(11)where 
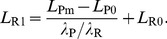
(12)


### Model of the signal delivered by the ORN population

The model is described in [69] with a few changes. Briefly, the response of each ORN stimulated at dose *C* is a spike train of latency *L* and interspike interval 1/*F* (Fig. 9C), where *L* is given by eq. 8 and *F* by eq. 4. The distributions of the parameters *F*_M_, *C*_1/2_, *n*, *L*_0_, *λ* and *L*_m_ (all Gaussian except *n* and *λ,* lognormal), their means μ and variances σ^2^ (see Figs. 6–8 E, F) and the correlations between parameters were determined from experimental data. This knowledge is expressed as the mean vector M and variance-covariance matrix Σ given in S6 Table. Each set of 6 parameter values characterizing an ORN was drawn from the 6-dimensional multinormal distribution defined by M and Σ. The model predicts the observed firing rates and latencies (no difference with observed distributions at doses from −2 to 2 using Kolmogorov-Smirnov test at level 1%, Fig. 9A, B). The spike trains of 7000 simulated ORNs were summated and the number of spikes fired by the population during each bin of 10 ms was counted. The number of Z7-12:Ac-responsive ORNs on the antenna (∼7000) was determined from the number of flagellar segments (∼90), the mean number of long sensilla trichodea per segment (∼80) and the number of Z7-12:Ac-responsive ORNs per sensilla (1).

## Supporting Information

Figure S1 **Determination of response latency and firing rate.** (**A**) Example of post-stimulus activity in an ORN at dose 2 log ng. Response latency *L* is the time elapsed from stimulus arrival *T*_t_ to the first spike in the response (horizontal arrow). (**B**) Firing rate function *f*(*t*) obtained by replacing each spike with a Gaussian curve of SD 50 ms. Response firing rate *F*_raw_ (vertical arrow) is the peak of *f*(*t*). (**C**) Example of post-stimulus activity in a PN at dose *C* = 0 log ng. (D) Firing rate corresponding to C, same representation as in B.
(EPS)

Figure S2 **Correlations between parameters of ORN dose-response curves.** Pearson's coefficients of correlation and their *p*-values (Student's *t* test for a transformation of the correlation) are given in the bottom right corner of each plot. Lognormally distributed parameters (*n*, *λ*) were normalized. (**A-C**) Parameters (*F*_M_, *C*_1/2_, *n*) fitted to ORN dose-firing rate curves. Plots of pairs for the same neuron; none of the 3 pairs are correlated. (**D-F**) Parameters (*L*_0_, *λ*, *L*_m_) fitted to ORN dose-latency curves; 2 pairs are correlated (*L*_0_-*λ* in D, *L*_0_-*L*_m_ in E). (**G-O**) Pairs of parameters with one from a *F*(*C*) curve and the other from the *L*(*C*) curve for the same neuron; a single of the 9 pairs is correlated (*C*_1/2_-*L*_0_ in J, see text). (**P**) Plot of characteristic *C*_0_ (threshold) *vs*. parameter *L*_m_ (minimum latency), not significantly correlated.
(EPS)

Figure S3 **Correlations between parameters of PN dose-response curves.** Same representation and tests as in S2 Figure. (**A–C**) Parameters (*F*_M_, *C*_1/2_, *n*) fitted to PN of *C*-*F* curves; none of the 3 pairs are correlated. (**D–F**) Parameters (*L*_0_, *λ*, *L*_m_) fitted to PN *C*-*L* curves; the 3 pairs are correlated. (**G–O**) Pairs of *F*-*C* and *L*-*C* parameters for the same neuron; none of the 9 pairs is correlated at level 1%. (**P**) Pair (*C*_0_, *L*_m_) is significantly correlated.
(EPS)

Figure S4 **Determination of irregularity and heterogeneity in time and amplitude of stimulus and ORN response.** (**A**) Overlaid PID signals following 14 repeated stimulations of 200 ms duration (black bar) with the *same* cartridge loaded with 100 µg of α-pinene. Small variations in time and amplitude illustrate stimulus *irregularity*. (**B**) Same as in (A) for 10 repeated stimulations with *different* identically prepared cartridges. Larger variations than in (A) illustrate stimulus *heterogeneity*. (**C**) Example of PID signal following a 200-ms stimulation (black line) with α-pinene at dose 5 log ng showing time *T* (arrow) elapsed between electrovalve opening and onset of PID signal. (**D**) Comparison of *T* PID in 10 series of 14 repetitions with the same dose (5 log ng) in a different cartridge in each series. Heterogeneity (difference between lines) is 3 times larger than irregularity (small fluctuations along a line). (**E**) Same PID signal as in A showing maximum signal amplitude *V* (arrow). (**F**) Comparison of *V* in 10 series of 14 repetitions with the same dose (5 log ng) in a different stimulus cartridge in each series. Heterogeneity is 4 times larger than irregularity. (**G**) Example of ORN response with response time *T* (arrow) following a 200-ms stimulation (black line) with Z7-12:Ac (2 log ng). (**H**) Comparison of *T* in 10 ORNs, each stimulated 14 times with the same dose (2 log ng) in a different cartridge for each ORN. Heterogeneity is 15% larger than irregularity. (**G**) Gaussian kernel estimate of the spike train shown in E with its maximum *F*_raw_ (arrow). (**H**) Comparison of *F*_raw_ in 10 ORNs, each stimulated 14 times with the same dose (2 log ng) in a different cartridge for each ORN. Heterogeneity is 3.75 times larger than irregularity.
(EPS)

Table S1 **Main symbols used in data analyses.**
(DOC)

Table S2 **Distributions of spontaneous firing rates** ***F*** **_sp_ (in AP/s).**
(DOC)

Table S3 **Distributions of fitted dose-firing rate properties of ORNs and PNs.**
(DOC)

Table S4 **Distributions of fitted dose-latency properties of ORNs and PNs.**
(DOC)

Table S5 **Correlations between fitted dose-response properties of ORNs and PNs.** In each cell: Pearson's coefficient of correlation and its *p*-value (Student's *t* test for a transformation of the correlation) after normalization of lognormally distributed properties (*n*, *λ, L*_M_). Significant correlations at level 0.01 shown in bold. Correlations between pairs of *F*-properties (upper right triangle, 15 values) and between pairs of *L*-properties (lower right triangle, 10 values) shown in roman. Correlations of *F*-properties with *L*-properties (upper right rectangle, 30 values) shown in italic.
(DOC)

Table S6 **Parameters of the multinormal distribution used to simulate the ORN population.**
(DOC)
