# Supplementary material for: Heterogeneity and Convergence of Olfactory First-Order Neurons Account for the High Speed and Sensitivity of Second-Order Neurons
Source: PLoS Comput Biol. 2014 Dec 4;10(12):e1003975. doi: 10.1371/journal.pcbi.1003975 (PMC4256018; doi:10.1371/journal.pcbi.1003975)
Supplement: Table S1 — Main symbols used in data analyses. (DOC) [file pcbi.1003975.s005.doc]

Table S1. Main symbols used in data analyses

| Variable | | Symbol | Definition |
| --- | --- | --- | --- |
| Observed | Stimulus | *C* | Dose, log_10_ of pheromone load in ng |
|  |  | *t* | Time after electrovalve onset of stimulation |
|  |  | *T* | Reaction time after electrovalve onset of stimulation |
|  |  | *T*_t_ | Transport time of stimulus to antenna = 180 ± SD 13 ms |
|  | Response | *F*_sp_ | Mean number of spikes per sec in spontaneous activity |
|  |  | *f*(*t*) | Firing rate (Gaussian kernel method) at time *t* |
|  |  | *F*_raw_ | Peak frequency of *f*(*t*) in a response to Z7-12:Ac |
|  |  | *F*_c_ | Peak of *f*(*t*) in a control stimulation (air, hexane) |
|  |  | *F* | Firing rate, pure olfactory component, *F* = *F*_raw_ − *F*_c_ |
|  |  | *L* | Latency, time *t* of first spike in a response , *L* = *T* − *T*_t_ |
| Fitted | Firing rate | *F*_M_ | Asymptotic maximum of *F* vs. *C* curve |
|  |  | *C*_1/2_ | Dose at half-maximum response *F*_M_/2 |
|  |  | *n* | Hill coefficient |
|  |  | *F*_0_ | Chosen threshold of *F* (*F*_0_ = 5 AP/s) |
|  |  | *C*_0_ | Dose at threshold *F* = *F*_0_, see eq. S6 |
|  |  | *C*_S_ | Dose at saturation *F* = *F*_M_ − *F*_0_ |
|  |  | *∆C* | Dynamic range of *F* vs. *C* curve, see eq. 7 |
|  | Latency | *λ* | Slope of *L* vs. *C* line |
|  |  | *L*_M_ | Maximum latency at dose *C*_0_ |
|  |  | *L*_0_ | Latency at *C =* 0 log ng (load 1 ng) |
|  |  | *L*_m_ | Minimum latency measured for a given neuron |
|  |  | *∆L* | Range of variation of *L*, *∆L = L*_M_ *- L*_m_ |
| Variability | Irregularity | *σ*_Sa_ | SD of stimulus irregularity, measured |
|  |  | *σ*_Ta_ | SD of observed response irregularity, measured |
|  |  | *σ*_Ba_ | SD of biological response irregularity, see eq. 1 |
|  | Hetero-geneity | *σ*_Sr_ | SD of stimulus heterogeneity, measured |
|  |  | *σ*_Tr_ | SD of observed response heterogeneity, measured |
|  |  | *σ*_Br_ | SD of biological response heterogeneity, see eq. 2 |
