# Supplementary material for: Heterogeneity and Convergence of Olfactory First-Order Neurons Account for the High Speed and Sensitivity of Second-Order Neurons
Source: PLoS Comput Biol. 2014 Dec 4;10(12):e1003975. doi: 10.1371/journal.pcbi.1003975 (PMC4256018; doi:10.1371/journal.pcbi.1003975)
Supplement: Table S2 — Distributions of spontaneous firing rates F sp (in AP/s). (DOC) [file pcbi.1003975.s006.doc]

**Table S2. Distributions of spontaneous firing rates *F*_sp_ (in AP/s)**

| Neuron type | Distribution^a^ | *μ* | *σ* | Min^b^ | Q_25_^c^ | Median | Q_75_^c^ | Max^b^ | IQ^d^ |
| --- | --- | --- | --- | --- | --- | --- | --- | --- | --- |
| ORN | logN | 1.23 | 0.71 | 0.5 | 2.1 | 3.4 | 5.5 | 10.6 | 3.4 |
| PN | logN | 2.47 | 0.52 | 4.7 | 8.3 | 11.8 | 16.8 | 31.0 | 8.5 |

^a^ logN: lognormal distribution of mean *µ* and standard deviation *σ* (Fig. 3C)

^b^ Minimum (respectively maximum) value of *F*_sp_ in the experimental sample

^c^ First (respect. third) quartile of fitted distribution, 25% (respect. 75%) *F*_sp_ values are smaller

^d^ Interquartile range of fitted distribution, IQ = Q_75_ – Q_25_
