# Supplementary material for: Heterogeneity and Convergence of Olfactory First-Order Neurons Account for the High Speed and Sensitivity of Second-Order Neurons
Source: PLoS Comput Biol. 2014 Dec 4;10(12):e1003975. doi: 10.1371/journal.pcbi.1003975 (PMC4256018; doi:10.1371/journal.pcbi.1003975)
Supplement: Table S3 — Distributions of fitted dose-firing rate properties of ORNs and PNs. (DOC) [file pcbi.1003975.s007.doc]

**Table S3. Distributions of fitted dose-firing rate properties of ORNs and PNs**

|  | Population | Statistics | Response properties | | | | | |
| --- | --- | --- | --- | --- | --- | --- | --- | --- |
|  |  |  | Parameters | | | Characteristics | | |
|  |  |  | *F*_M_ | *C*_1/2_ | *n* | *C*_0_ | *C*_S_ | Δ*C* |
| Distribution of  dose-firing rate  properties | ORN^a^ | Unit | AP/s | log ng | (log ng)^-1^ | log ng | log ng | log unit |
|  |  | Type | N | N | logN | N | N | N |
|  |  | *N* | 38 | 38 | 38 | 38 | 38 | 38 |
|  |  | *μ* | 162 | 1.15 | -0.27 | -0.93 | 3.2 | 4.1 |
|  |  | *σ* | 34 | 0.57 | 0.35 | 1.0 | 1.1 | 1.8 |
|  |  | *p* | 0.74 | 0.95 | 0.29 | 0.60 | 0.25 | 0.06 |
|  | PN^b^ | Type | N | N | logN | N | N | N |
|  |  | *N* | 37 | 37 | 37 | 37 | 37 | 37 |
|  |  | *μ* | 67 | -1.5 | 0.04 | -2.8 | -0.29 | 2.4 |
|  |  | *σ* | 37 | 0.76 | 0.82 | 0.88 | 1.5 | 1.8 |
|  |  | *p* | 0.75 | 0.94 | 0.09 | 0.97 | 0.86 | 0.89 |
|  | ORN/PN^c^ | *P* | <10^-13^ | <10^-16^ | 0.03 | <10^-9^ | <10^-15^ | <10^-3^ |
| Statistics of  dose-firing rate  properties | ORN^d^ | Q_10_ | 118 | 0.54 | 0.52 | -2.0 | 2.2 | 2.7 |
|  |  | Q_25_ | 136 | 0.72 | 0.70 | -1.3 | 2.5 | 3.2 |
|  |  | Median | 162 | 1.1 | 0.81 | -0.82 | 2.8 | 3.6 |
|  |  | Q_75_ | 185 | 1.5 | 0.92 | -0.26 | 3.6 | 4.5 |
|  |  | Q_90_ | 209 | 1.9 | 1.10 | 0.20 | 4.2 | 5.6 |
|  |  | IQ | 49 | 0.82 | 0.22 | 1.0 | 1.2 | 1.3 |
|  |  | IQ/med | 0.30 | - | 0.27 | - | - | 0.37 |
|  | PN^e^ | Q_10_ | 27 | -2.3 | 0.44 | -3.8 | -1.7 | 0.28 |
|  |  | Q_25_ | 44 | -2.0 | 0.57 | -3.4 | -1.4 | 1.0 |
|  |  | Median | 62 | -1.5 | 0.79 | -2.7 | 0.0 | 2.5 |
|  |  | Q_75_ | 83 | -1.1 | 2.1 | -2.2 | 0.65 | 3.5 |
|  |  | Q_90_ | 109 | -0.29 | 3.9 | -1.6 | 1.2 | 4.7 |
|  |  | IQ | 39 | 0.86 | 1.5 | 1.1 | 2.0 | 2.5 |
|  |  | IQ/med | 0.62 | - | 1.9 | - | - | 1.0 |

^a^ ORN response characteristics based on dose-response curves: type (normal N or lognormal logN) and parameters of the distribution (mean *µ*, standard-deviation *σ*); *p* value of Kolmogorov-Smirnov test comparing empirical distribution to theoretical. All differences are non-significant.

^b^ PN characteristics. Same description and test as for ORNs. All differences are non-significant.

^c^ *P* value of Kolmogorov-Smirnov test comparing the ORN and PN distributions (test of null hypotheses ORN = PN against ORN ≠ PN). All tests significant at level 1% except for *n*.

^d, e^ Q_x_, quantiles of the observed values, x% of values are smaller. IQ, interquartile range, IQ = Q_75_ – Q_25_. IQ/med, ratio IQ/median (meaningless for dose data, *C*_0_, *C*_1/2_, *C*_S_).
