# Supplementary material for: Heterogeneity and Convergence of Olfactory First-Order Neurons Account for the High Speed and Sensitivity of Second-Order Neurons
Source: PLoS Comput Biol. 2014 Dec 4;10(12):e1003975. doi: 10.1371/journal.pcbi.1003975 (PMC4256018; doi:10.1371/journal.pcbi.1003975)
Supplement: Table S4 — Distributions of fitted dose-latency properties of ORNs and PNs. (DOC) [file pcbi.1003975.s008.doc]

**Table S4. Distributions of fitted dose-latency properties of ORNs and PNs**

|  | Population | Statistics | Response properties | | | | |
| --- | --- | --- | --- | --- | --- | --- | --- |
|  |  |  | Parameters | | | Characteristics | |
|  |  |  | *L*_0_ | *λ* | *L*_m_ | *L*_M_ | *ΔL* |
| Distribution  of  dose-latency  response  properties | ORN^a^ | Unit | ms | ms/(log ng) | ms | ms | ms |
|  |  | Type | N | logN | N | logN | N |
|  |  | *N* | 38 | 38 | 38 | 38 | 38 |
|  |  | *µ* | 158 | 2.9 | 67 | 5.1 | 109 |
|  |  | *σ* | 62 | 0.85 | 30 | 0.39 | 55 |
|  |  | *P* | 0.82 | 0.12 | 0.87 | 0.89 | 0.44 |
|  | PN^b^ | Type | N | logN | N | logN | N |
|  |  | *N* | 44 | 44 | 44 | 41 | 41 |
|  |  | *μ* | 64 | 3.1 | 32 | 4.7 | 87 |
|  |  | *σ* | 36 | 1.3 | 40 | 0.54 | 75 |
|  |  | *P* | 0.79 | 0.02 | 0.59 | 0.97 | 0.08 |
|  | ORN/PN^c^ | *P* | <10^-9^ | 0.01 | <10^-2^ | <10^-4^ | <10^-2^ |
| Statistics  of  dose-latency  response  properties | ORN^d^ | Q_10_ | 87 | 7.3 | 32 | 114 | 47 |
|  |  | Q_25_ | 121 | 16 | 43 | 137 | 79 |
|  |  | Median | 157 | 24 | 64 | 164 | 104 |
|  |  | Q_75_ | 192 | 29 | 89 | 208 | 138 |
|  |  | Q_90_ | 248 | 43 | 115 | 282 | 188 |
|  |  | IQ | 72 | 13 | 47 | 71 | 59 |
|  |  | IQ/med | 0.46 | 0.53 | 0.73 | 0.43 | 0.57 |
|  | PN^e^ | Q_10_ | 19 | 6.7 | 8 | 59 | 36 |
|  |  | Q_25_ | 41 | 11 | 25 | 79 | 43 |
|  |  | Median | 62 | 16 | 44 | 107 | 64 |
|  |  | Q_75_ | 89 | 25 | 63 | 167 | 107 |
|  |  | Q_90_ | 117 | 55 | 83 | 245 | 185 |
|  |  | IQ | 48 | 15 | 38 | 88 | 64 |
|  |  | IQ/med | 0.78 | 0.94 | 0.88 | 0.82 | 1.00 |

^a^ ORN response characteristics based on dose-response curves: type (normal N or lognormal logN) and parameters of the distribution (mean *µ*, standard-deviation *σ*); *p* value of Kolmogorov-Smirnov test comparing empirical distribution to theoretical. All differences are non-significant.

^b^ PN characteristics. Same description and test as for ORNs. All differences are non-significant.

^c^ *P* value of Kolmogorov-Smirnov test comparing the ORN and PN distributions (test of null hypotheses ORN = PN against ORN ≠ PN). Tests significant at level 1% except for λ and *L*_M_.

^d, e^ Q_x_, quantiles of the observed values, x% of values are smaller. IQ, interquartile range, IQ = Q_75_ – Q_25_. IQ/med, ratio IQ/median.
