# Supplementary material for: Heterogeneity and Convergence of Olfactory First-Order Neurons Account for the High Speed and Sensitivity of Second-Order Neurons
Source: PLoS Comput Biol. 2014 Dec 4;10(12):e1003975. doi: 10.1371/journal.pcbi.1003975 (PMC4256018; doi:10.1371/journal.pcbi.1003975)
Supplement: Table S5 — Correlations between fitted dose-response properties of ORNs and PNs. In each cell: Pearson's coefficient of correlation and its p-value (Student's t test for a transformation of the correlation) after normalization of lognormally distributed properties (n, λ, L M). Significant correlations at level 0.01 shown in bold. Correlations between pairs of F-properties (upper right triangle, 15 values) and between pairs of L-properties (lower right triangle, 10 values) shown in roman. Correlations of F-properties with L-properties (upper right rectangle, 30 values) shown in italic. (DOC) [file pcbi.1003975.s009.doc]

Table S5. Correlations between fitted dose-response properties of ORNs and PNs

| ORN | Firing rate properties | | | | | | Latency properties | | | | |
| --- | --- | --- | --- | --- | --- | --- | --- | --- | --- | --- | --- |
|  | Parameters | | | Characteristics | | | Parameters | | | Characteristics | |
|  | *F*_M_ | *C*_1/2_ | ln *n* | *C*_0_ | *C*_s_ | *ΔC* | *L*_0_ | ln λ | *L*_m_ | ln *L*_M_ | *ΔL* |
| *F*_M_ | 1  0 | 0.15  0.37 | -0.27  0.10 | -0.32  0.05 | 0.41  0.01 | **0.44**  **10^-2^** | *-0.29*  *0.07* | *-0.22*  *0.18* | *-0.26*  *0.11* | *-0.20*  *0.24* | *-0.10*  *0.56* |
| *C*_1/2_ |  | 1  0 | -0.17  0.30 | 0.40  0.01 | **0.67**  **10^-5^** | 0.23  0.17 | ***0.50***  ***10^-2^*** | *0.24*  *0.14* | *0.23*  *0.17* | *0.28*  *0.09* | *0.27*  *0.10* |
| ln *n* |  |  | 1  0 | **0.79**  **10^-8^** | **-0.81**  **10^-9^** | **-0.95**  **10^-18^** | *-0.01*  *0.93* | *0.08*  *0.63* | *0.08*  *0.64* | *-0.25*  *0.14* | *-0.40*  *0.01* |
| *C*_0_ |  |  |  | 1  0 | **-0.41**  **10^-2^** | **-0.80**  **10^-8^** | *0.31*  *0.06* | *0.21*  *0.22* | *0.26*  *0.11* | *-0.06*  *0.71* | *-0.23*  *0.16* |
| *C*_s_ |  |  |  |  | 1  0 | **0.88**  **10^-12^** | *0.25*  *0.13* | *0.07*  *0.66* | *0.01*  *0.94* | *0.33*  *0.04* | ***0.46***  ***10^-2^*** |
| *Δ*C |  |  |  |  |  | 1  0 | *0*  *1.00* | *-0.06*  *0.72* | *-0.13*  *0.43* | *0.25*  *0.13* | ***0.43***  ***10^-2^*** |
| *L*_0_ |  |  |  |  |  |  | 1  0 | **0.59**  **10^-3^** | **0.75**  **10^-7^** | **0.86**  **10^-11^** | **0.64**  **10^-4^** |
| ln *λ* |  |  |  |  |  |  |  | 1  0 | 0.09  0.58 | **0.74**  **10^-7^** | **0.79**  **10^-8^** |
| *L*_m_ |  |  |  |  |  |  |  |  | 1  0 | **0.56**  **10^-3^** | 0.11  0.51 |
| ln *L*_M_ |  |  |  |  |  |  |  |  |  | 1  0 | **0.85**  **10^-11^** |
| *ΔL* |  |  |  |  |  |  |  |  |  |  | 1  0 |

| PN | *F*_M_ | *C*_1/2_ | ln *n* | *C*_0_ | *C*_s_ | *ΔC* | *L*_0_ | ln *λ* | *L*_m_ | ln *L*_M_ | *ΔL* |
| --- | --- | --- | --- | --- | --- | --- | --- | --- | --- | --- | --- |
| *F*_M_ | 1  0 | 0.28  0.09 | -0.37  0.02 | **-0.42**  **10^-2^** | **0.54**  **10^-3^** | **0.63**  **10^-4^** | *-0.12*  *0.48* | *0.18*  *0.29* | *-0.22*  *0.21* | *0.22*  *0.19* | ***0.43***  ***10^-2^*** |
| *C*_1/2_ |  | 1  0 | **-0.54**  **10^-3^** | 0.37  0.02 | **0.85**  **10^-10^** | **0.51**  **10^-2^** | *0.15*  *0.39* | *0.17*  *0.33* | *0.11*  *0.52* | *0.18*  *0.28* | *0.18*  *0.28* |
| ln *n* |  |  | 1  0 | 0.45  10^-2^ | **-0.83**  **10^-9^** | **-0.88**  **10^-12^** | *0.30*  *0.07* | *-0.13*  *0.45* | *0.35*  *0.04* | *-0.07*  *0.69* | *-0.21*  *0.23* |
| *C*_0_ |  |  |  | 1  0 | -0.18  0.28 | **-0.61**  **10^-4^** | *0.38*  *0.02* | *-0.07*  *0.68* | ***0.46***  ***10^-2^*** | *-0.07*  *0.69* | *-0.27*  *0.11* |
| *C*_s_ |  |  |  |  | 1  0 | **0.89**  **10^-12^** | *-0.07*  *0.67* | *0.22*  *0.20* | *-0.16*  *0.35* | *0.23*  *0.17* | *0.36*  *0.03* |
| *ΔC* |  |  |  |  |  | 1  0 | *-0.24*  *0.16* | *0.21*  *0.23* | *-0.35*  *0.04* | *0.22*  *0.20* | *0.41*  *0.01* |
| *L*_0_ |  |  |  |  |  |  | 1  0 | **0.39**  **10^-2^** | **0.92**  **10^-18^** | **0.64**  **10^-5^** | 0.23  0.14 |
| ln *λ* |  |  |  |  |  |  |  | 1  0 | 0.16  0.31 | **0.59**  **10^-4^** | **0.59**  **10^-4^** |
| *L*_m_ |  |  |  |  |  |  |  |  | 1  0 | **0.49**  **10^-2^** | 0.05  0.74 |
| ln *L*_M_ |  |  |  |  |  |  |  |  |  | 1  0 | **0.85**  **10^-11^** |
| *ΔL* |  |  |  |  |  |  |  |  |  |  | 1  0 |

In each cell: Pearson’s coefficient of correlation and its *p*-value (Student’s *t* test for a transformation of the correlation) after normalization of lognormally distributed properties (*n*, *λ, L*_M_). Significant correlations at level 0.01 shown in bold. Correlations between pairs of *F*-properties (upper right triangle, 15 values) and between pairs of *L*-properties (lower right triangle, 10 values) shown in roman. Correlations of *F*-properties with *L*-properties (upper right rectangle, 30 values) shown in italic.
