# Supplementary material for: Heterogeneity and Convergence of Olfactory First-Order Neurons Account for the High Speed and Sensitivity of Second-Order Neurons
Source: PLoS Comput Biol. 2014 Dec 4;10(12):e1003975. doi: 10.1371/journal.pcbi.1003975 (PMC4256018; doi:10.1371/journal.pcbi.1003975)
Supplement: Table S6 — Parameters of the multinormal distribution used to simulate the ORN population. (DOC) [file pcbi.1003975.s010.doc]

**Table S6. Parameters of the multinormal distribution used to simulate the ORN population**

| Mean vector *M* | | | | | | |
| --- | --- | --- | --- | --- | --- | --- |
|  | *F*_M_ | *C*_1/2_ | ln *n* | *L*_0_ | ln *λ* | *L*_m_ |
| µ | 161.60 | 1.15 | -0.27 | 157.91 | 2.93 | 67.46 |
|  | | | | | | |
| Variance-covariance matrix *Σ* | | | | | | |
|  | *F*_M_ | *C*_1/2_ | ln *n* | *L*_0_ | ln *λ* | *L*_m_ |
| *F*_M_ | 1158.36 | 2.87 | -2.59 | -617.71 | -8.79 | -266.62 |
| *C*_1/2_ | 2.87 | 0.32 | -0.02 | 17.50 | 0.08 | 3.85 |
| ln *n* | -2.59 | -0.02 | 0.12 | 0 | 0 | 0.53 |
| *L*_0_ | -617.71 | 17.50 | 0 | 3788.39 | 28.40 | 1385.50 |
| ln *λ* | -8.79 | 0.08 | 0 | 28.40 | 0.72 | 2.95 |
| *L*_m_ | -266.62 | 3.85 | 0.53 | 1385.50 | 2.95 | 899.53 |
